# The Biosynthesis of the Benzoxazole in Nataxazole Proceeds via an Unstable Ester and has Synthetic Utility

**DOI:** 10.1002/anie.201915685

**Published:** 2020-02-11

**Authors:** Haigang Song, Cong Rao, Zixin Deng, Yi Yu, James H. Naismith

**Affiliations:** ^1^ Key Laboratory of Combinatorial Biosynthesis and Drug Discovery (Ministry of Education) School of Pharmaceutical Sciences Wuhan University 185 East Lake Road Wuhan 430071 P. R. China; ^2^ Division of Structural Biology Wellcome Centre for Human Genetics Roosevelt Drive Oxford OX3 7BN UK; ^3^ The Research Complex at Harwell Harwell Campus OX11 0FA UK; ^4^ The Rosalind Franklin Institute Harwell Campus OX11 0FA UK

**Keywords:** benzoxazole, enzyme catalysis, heterocycles, structural biology, structure–activity relationships

## Abstract

Heterocycles, a class of molecules that includes oxazoles, constitute one of the most common building blocks in current pharmaceuticals and are common in medicinally important natural products. The antitumor natural product nataxazole is a model for a large class of benzoxazole‐containing molecules that are made by a pathway that is not characterized. We report structural, biochemical, and chemical evidence that benzoxazole biosynthesis proceeds through an ester generated by an ATP‐dependent adenylating enzyme. The ester rearranges via a tetrahedral hemiorthoamide to yield an amide, which is a shunt product and not, as previously thought, an intermediate in the pathway. A second zinc‐dependent enzyme catalyzes the formation of hemiorthoamide from the ester but, by shuttling protons, the enzyme eliminates water, a reverse hydrolysis reaction, to yield the benzoxazole and avoids the amide. These insights have allowed us to harness the pathway to synthesize a series of novel halogenated benzoxazoles.

## Graphical abstract

**Benzoxazole biosynthesis**, a key step in the biosynthesis of the antitumor agent nataxazole, proceeds via a hemiorthoamide intermediate that arises from an unstable ester. NatL2 catalyzes the formation of this ester that spontaneously rearranges into an off‐pathway amide. In the presence of NatAM, the ester is converted to benzoxazole via a hemiorthoamide intermediate. These enzymes are used to synthesize a series of novel halogenated benzoxazoles.

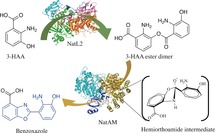

## Introduction

**Figure 1:**
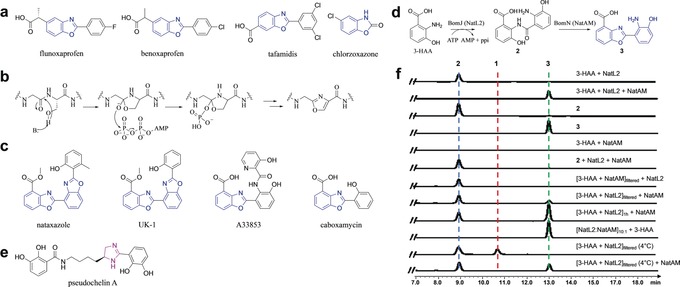
Benzoxazoles and formation by NatL2 and NatAM. a) Currently marketed drugs containing benzoxazole (blue). b) Mechanism for biosynthesis of oxazoles in RiPPs involving a hemiorthoamide intermediate. c) Benzoxazole‐containing natural products. d) The proposed benzoxazole biosynthetic pathway employed by BomJ and BomN proceeds via an amide intermediate **2**. NatL2 and NatAM (homologues of BomJ and BomN) are thought to operate the same steps in the nataxazole biosynthesis. e) Pseudochelin A has an imidazoline ring (purple) whose formation is thought to involve an amidohydrolase (MxcM). f) HPLC profiles of in vitro assays show NatL2 and NatAM together yield expected product **3**. Filtering at 4 °C (to remove NatL2) reveals an intermediate, compound **1**, isobaric to the amide. **1** is converted to product **3** by the addition of NatAM.

Over 80 % of currently prescribed small molecule drugs contain a heterocyclic ring.^1^ Heterocycles are rigid molecules that can form hydrogen bonds as well as hydrophobic interactions, moreover they can be modified to adjust lipophilicity, polarity, aqueous solubility, and selectivity. Oxazoles (and oxazolines) are very common heterocycles and are found isolated or conjugated to aromatic rings, for example, with benzene, known as benzoxazoles. Benzoxazoles are frequently found in pharmaceutically active compounds^2^ and natural products for example in plants (neosalvianen, salvianen, and salvianan);^3^ marine sponges (nakijinol,^4^ pseudopteroxazole,^5^ and ileabethoxazole)^6^ as well as bacteria (UK‐1,^7^ caboxamycin,^8^ A33853,^9^ calcimycin,^10^ nataxazole,^11^ and AJI956).^12^ Benzoxazole compounds are prescribed as anticancers, anti‐inflammatories (flunoxaprofen,^13^ benoxaprofen^14^), antibacterial (calcimycin,^15^ boxazomycin^16^), and muscle relaxant (chlorzoxazone^17^). Tafamidis (Figure 1 a) is the only approved medicine to delay progression of transthyretin familial amyloid polyneuropathy.^18^, ^19^

Unconjugated oxazoles are mostly produced by ribosomally synthesized post‐translationally modified peptides (RiPPs) synthesis and by non‐ribosomal peptides (NRPs) synthesis.^20^, ^21^ Oxazoles in both families derive from nucleophilic attack of the hydroxyl group of Ser(Thr) upon amide bonds^20^, ^22^ through a common hemiorthoamide intermediate (Figure 1 b), but via distinct enzymatic routes.^23^, ^24^, ^25^, ^26^, ^27^, ^28^, ^29^, ^30^, ^31^, ^32^, ^33^, ^34^ Two polyketide synthase–NRPS hybrid natural products, oxazolomycin and conglobatin, have been proposed to utilize an N‐type ATP pyrophosphohydrolase to form oxazoles.^35^, ^36^ None of these enzymes have been reported to catalyze the formation of benzoxazole and such activity would seem unlikely based on their known substrates.

The biosynthetic gene clusters for benzoxazole‐containing natural products calcimycin (*Streptomyces chartreusis NRRL 3882*), nataxazole (*S*. sp. Tü 6176), A33853 (*S*. sp. NRRL12068), and caboxamycin (*S*. sp. NTK 937), have been identified (Figure 1 c).^37^, ^38^, ^39^, ^40^ Sequence analysis failed to identify any homologue to the enzymes in RiPPs or NRPs or an N‐type ATP pyrophosphohydrolase. In vivo knock‐outs in the A33853 biosynthetic cluster were used to propose a pathway in which 3‐hydroxyanthranilic acid (3‐HAA)^38^ undergoes dimerization by amide formation catalyzed by BomJ (putative phenylacetate CoA ligase; Figure 1 d). This amide is then rearranged by the intramolecular attack of a hydroxyl and ring closure catalyzed by BomN (putative amidohydrolase)^38^ (Figure 1 d). Since the gene clusters for nataxazole and caboxamycin all contain the homologue of the *bomN* amidohydrolase, the synthesis of the benzoxazole is expected to be the same in each case.^40^

Acyl‐CoA ligases, which include the structurally characterized phenylacetate‐coenzyme A ligase (PaaK), catalyze the formation of CoA thioester derivatives by two half reactions: adenylation of the carboxylic acid, and subsequent thioesterification. Non‐thioesterifying acyl‐CoA ligases, are known but rare, found only in fatty acyl‐AMP ligases (FAAL)^41^, ^42^, ^43^ and PtmA1.^44^ The FAAL enzymes block binding of CoA through an insertion in the C‐terminal domain (FAAL‐specific insertion) and instead recognize 4′‐phosphopantetheine from acyl carrier proteins. BomJ would represent the first amide‐forming PaaK‐type enzyme. BomN is of interest since amidohydrolases catalyze hydrolysis not ring formation. Although the distantly related MxcM enzyme was reported to participate in imidazoline ring formation in pseudochelin biosynthesis^45^ (Figure 1 e).

We have determined the structures of both NatL2 (homologue of BomJ) and NatAM (homologue of BomN) from the antitumor nataxazole (Figure 1 d) pathway. We show NatL2 catalyzes ester not, as previously reported, amide formation; the amide is a shunt product. NatAM catalyzes benzoxazole from this ester by a mechanism that we propose involves shuttling of protons to control the fate of a hemiorthoamide intermediate. Guided by these insights, we have used the enzymes to make new halogenated benzoxazoles.

## Results and Discussion

### Biochemistry of NatL2 and NatAM

NatL2 and NatAM were expressed and purified as N‐terminal hexahistidine‐tagged fusion proteins in *E. coli* and *S*. *albus, respectively* (NatAM expressed in *E. coli* was insoluble). HPLC analysis detected the benzoxazole, (**3,** 2‐(2‐amino‐3‐hydroxyphenyl)benzoxazole‐4‐carboxylic acid) in the presence of NatL2, NatAM, ATP, 3‐HAA, and MgCl_2_ (Figure 1 f), in agreement with the BomJ/BomN study.^38^ High‐resolution electrospray ionization mass spectrometry (HR‐ESIMS; *m*/*z* 271.0716 [*M*+H]^+^, Supporting Information, Figure S1), ^1^H‐NMR and ^13^C‐NMR spectroscopy (Supporting Information, Figure S2) confirmed **3**. When NatAM, NatL2, ATP or 3‐HAA were individually omitted, **3** was not detected.

The amide “intermediate”^38^ **2,** (2‐amino‐3‐((2‐carboxy‐6‐hydroxyphenyl)carbamoyl)benzoic acid), was detected (HR‐MS/MS, ^1^H‐NMR, and ^13^C‐NMR, Supporting Information, Figures S1 and S2) following incubation of NatL2 with ATP, 3‐HAA, and MgCl_2_. We incubated **2** with NatAM but no **3** or consumption of **2** were detected (Figure 1 f) despite addition of various combinations of ATP, AMP, ZnCl_2_, 3‐HAA, and pyrophosphate. Purified **2** was incubated with NatL2 and NatAM but no consumption of **2** or synthesis of **3** were observed (Figure 1 f). ITC titration and native gels showed no evidence for complex formation (Supporting Information, Figure S3). Two parallel 60 minute incubations were performed in the presence of ATP, 3‐HAA, and MgCl_2_ adding either NatAM or NatL2 then filtering through a 10 kDa membrane to remove protein. NatAM was added to the filtrate from the NatL2 reaction and NatL2 to NatAM filtrate. LC‐MS analysis detected both **2** and **3** when NatAM was added to the filtrate of NatL2. As expected only **2** was detected when NatL2 was added to the filtrate of NatAM (Figure 1 f). We suspected that NatL2 produced an undetected compound that is the substrate for NatAM but that this compound has converted to **2**. We therefore incubated ATP, 3‐HAA, MgCl_2_, and NatL2 but the reaction was filtered at 4 °C. LC‐MS shows a new peak, compound **1** which has the same weight and fragmentation pattern as **2** (Supporting Information, Figure S1) indicating a very close molecular relationship. Upon standing, **1** converts to the amide **2** (Supporting Information, Figure S3), whilst NatAM addition yields the product **3** (Figure 1 f). Attempts to isolate a sufficient amount of **1** for NMR analysis failed due to its conversion to **2**.

### Structural Biology of NatL2

**Figure 2:**
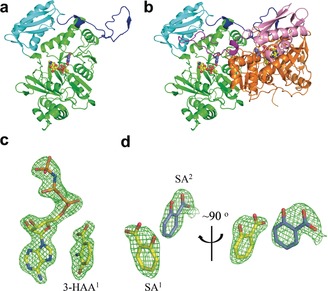
Structure biology of NatL2. a) The NatL2 N‐terminal domain, C‐terminal domain, and C‐terminal extension are coloured green, cyan, and blue, respectively. AMP and 3‐HAA^1^ are shown as spacefill (O, red; N, blue; P, orange; C, yellow, except in 3HAA^2^ where carbons are in marine). (b) NatL2 dimer (N‐terminal domain, C‐terminal domain, and C‐terminal extension are in orange, pink, and purple in second monomer). c) Simulated‐annealing Fo‐Fc omit density contoured at 3 σ showing AMPPNP and 3‐HAA^1^. d) SA^1^ has displaced 3‐HAA^1^ and AMP. Orthogonal views of the simulated‐annealing Fo‐Fc omit density contoured at 3 σ showing SA^1^ and SA^2^.

We determined the 1.9 Å‐resolution crystal structure of NatL2 in the presence of ATP (Figure 2 a and Supporting Information, Table S3). NatL2 consists of two subunits in one asymmetric unit that PISA^46^ indicates form a homodimer (Figure 2 b). The individual monomers of NatL2 are structurally equivalent and can be superimposed with a root mean square deviation (rmsd) of 0.348 Å over 420 Cα atoms. Residues 5–436 in chain A and 1–322, 327–436 in chain B were traced. Residual electron density in both monomers was best modelled as AMP not ATP. NatL2 has an N‐terminal domain (1–320), a C‐terminal domain (326–404), and a C‐terminal extension (405–436). The N‐terminal domain, which binds AMP (Figure 2 a), forms an αβαβα structure with an additional β‐hairpin. The C‐terminal domain starts with a β‐hairpin followed by a three‐stranded β‐sheet flanking three α‐helices (Figure 2 a). A tetrahedrally coordinated zinc (Cys248, Cys308, Cys310, His254) is found in the N‐terminal domain over 20 Å away from AMP (Supporting Information, Figure S4). Treatment with EDTA abolished its activity, which was restored by adding Zn^2+^ (Supporting Information, Figure S4) leading to the conclusion the Zn^2+^ plays an essential structural role.

NatL2 resembles acetyl‐CoA synthetases, with an rmsd of 1.9 Å over 362 Cα atoms with PaaK from *Burkholderia cenocepacia* (PDB 2Y27, 2Y4N; 2.1 Å over 368 Cα for a putative acyl‐CoA ligase from *Bacteroides Thetaiotaomicron* (PDB 4RVL)). The Zn^2+^ is a unique feature of NatL2. The adenine and ribose rings of AMP superimpose in the structures; the α‐phosphate is displaced by 1.2 Å. In NatL2, the adenine ring is sandwiched between the main chain peptide bonds of Ala212 to Pro214 on one side and Tyr236 on the other. In broad terms, the binding site of AMP in NatL2 is conserved in PaaK. However, in NatL2, the α‐phosphate makes a salt bridge with Lys418 from the other monomer (Supporting Information, Figure S6), whilst in PaaK (PDB 2Y27) Lys422 in the same monomer plays this role. The salt bridge between lysine and the α‐phosphate is a feature of these enzymes,^47^ indicating the dimeric arrangement is functional. The C‐terminal extension (405–436) is a novel feature of NatL2 and has an extended strand followed by a turn and a shorter strand that reaches across to the other monomer filling a pocket in other monomer's C‐terminal domain below the AMP binding site (Supporting Information, Figure S5). In Paak, this pocket is filled by a shorter stretch of amino acids from within the same monomer which folds into two β‐turns (Supporting Information, Figure S5), reminiscent of strand exchange phenomena.^48^ As a result of this “cross over” in NatL2, the extension and C‐terminal domain from the other subunit are linked by three salt bridges, twelve hydrogen bonds, and extensive hydrophobic contacts.

**Figure 3:**
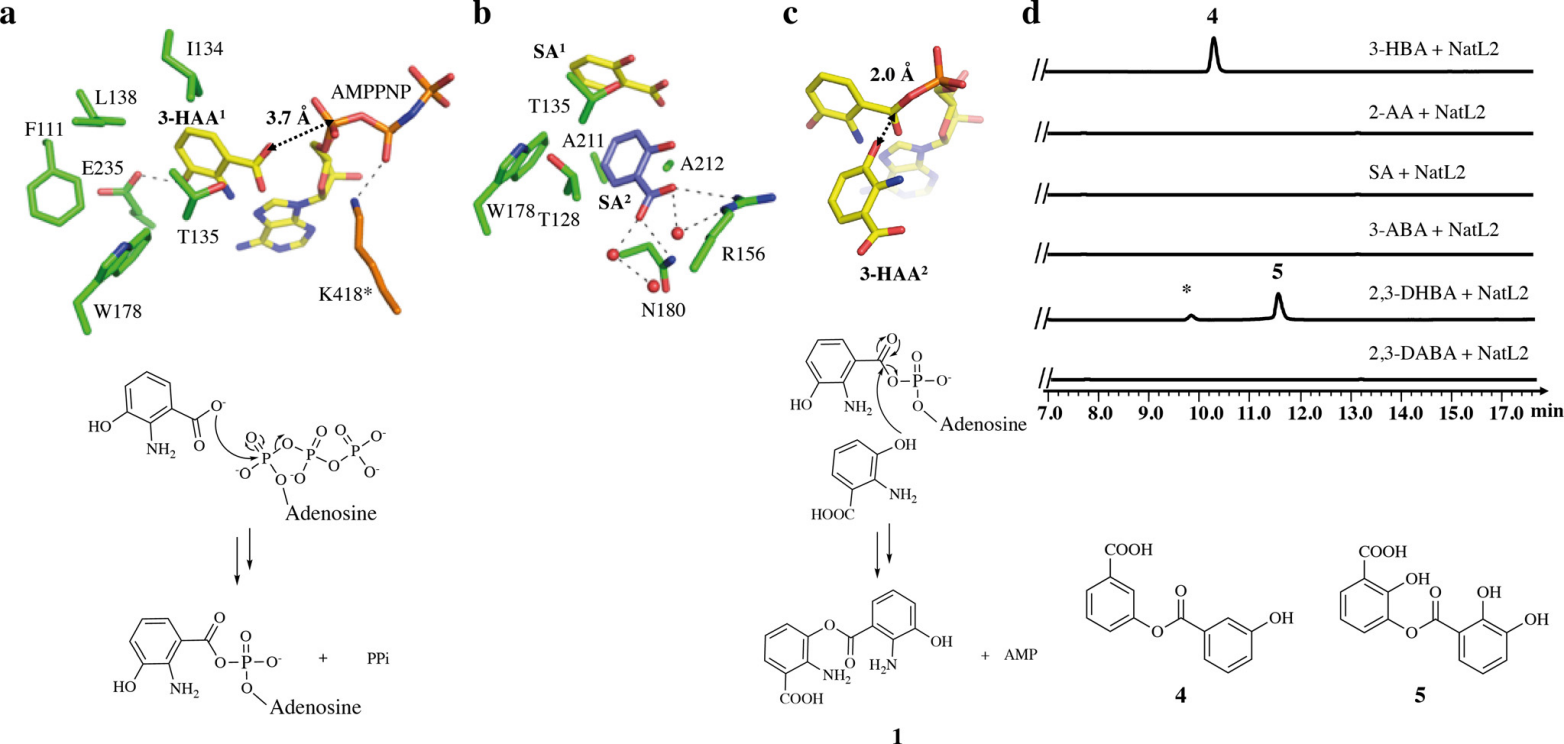
The mechanism of the two half reactions of NatL2. a) NatL2 complex of 3‐HAA^1^ with AMPPNP. This complex is a model for the first half reaction, adenylation of the carboxylate. Residues from monomer A are shown with the carbons in green, whist the carbons of Lys418 (highlighted with a *) from the other monomer are in orange. b) NatL2 complex of SA^1^ and SA^2^. c) In silico modelling of the second half reaction, formation of ester **1**, with 3‐HAA^2^ positioned in the second pocket (based on SA^2^ location) and 3‐HAA^1^‐adenylate (based on 3‐HAA1 AMP complex). d) NatL2 catalyzes ester **4** and **5** formation from 3‐HBA and 2,3‐DHBA , respectively. No product is observed when there is no hydroxyl group at the 3‐position. The 2,3‐DHBA reaction has an unknown impurity, marked with a *.

These crystals were soaked with AMPPNP and the resulting complex was essentially identical (α‐phosphate moved 1.0 Å) (Supporting Information, Figure S6). The β‐phosphate of AMPPNP interacts with the side chains of Ser87, Ser88, and Thr239, and backbone amides of Ser88 and Thr239, while the γ‐phosphate has fewer interactions making hydrogen bonds with a water molecule and the side chain of Ser88. There was no electron density for bound Mg^2+^. We soaked the AMP crystals with both AMPPNP and 3‐HAA overnight and saw clear density for both molecules (Figure 2 c). The adenosine and ribose rings of AMPPNP in this complex overlap with their positions in the AMPPNP binary complex (Supporting Information, Figure S6). The α‐ and β‐phosphates in the AMPPNP:3‐HAA ternary complex have however shifted and overlap with the β‐ and γ‐phosphates in the AMPPNP. In the AMPPNP:3‐HAA ternary complex, the γ‐phosphate salt bridges to Lys62 and Arg68 (Figure 3 a). The 3‐HAA molecule binds in a pocket adjacent to the AMPPNP positioning one oxygen of the carboxylate group 3.3 Å from the α‐phosphate with an angle of attack of 167° (O_carboxylate_–Pα–O_phosphoanhydride_) consistent with adenylation of the carboxylate. Thr239 hydrogen bonds to both α‐phosphate and carboxylate oxygen atoms. The hydroxyl group of 3‐HAA is hydrogen bonded to Glu235 whilst the amino group makes a hydrogen bond to N7 of the adenosine ring. The benzene ring stacks against a stretch of the main chain of Gly237 and Ser238. This complex is model for the Michealis complex of the first half (adenylation) reaction (Figure 3 a).

### Identifying the Product of NatL2

We soaked NatL2‐AMP crystals with 3‐HAA and obtained electron density for 3‐HAA and AMP (Supporting Information, Figure S7). 3‐HAA (denoted as 3‐HAA^1^) binds almost identically to the molecule in the AMPPNP:3‐HAA complex. There is residual electron density suggesting that we could fit a second molecule of 3‐HAA (3‐HAA^2^) bound adjacent to the 3‐HAA^1^ molecule; the orientation suggested that the hydroxyl not the amine would attack the 3‐HAA^1^‐adenylate (Supporting Information, Figure S7). We reasoned the low quality of the residual density arose from the crowding of carboxylate of 3‐HAA^1^, hydroxyl of 3‐HAA^2^ and the α‐phosphate of AMP. We therefore soaked the co‐crystallized AMP:3HAA crystals with salicylic acid (SA), which lacks the hydroxyl at the *meta* position. In subunit A, two molecules of SA are bound but no AMP (Figure 2 d). One SA molecule, denoted SA^1^ has replaced 3‐HAA^1^ (Figure 3 b). and occupies the same pocket with differences in orientation (Supporting Information, Figure S7). A second molecule of SA, denoted SA^2^ is unambiguously located (Figure 2 d) where we had tentatively identified 3‐HAA^2^. The pocket is formed by Thr128, Ala211, Ala212, Thr135, and Trp178. The carboxylate of SA^2^ makes a salt bridge with Arg156 that orients the molecule. A simple in silico change of the SA^2^ molecule to 3‐HAA^2^ molecule and combination with the 3‐HAA^1^‐AMP adenylate allows creation a model for the second reaction, the attack of hydroxyl (not the amine) of 3‐HAA^2^ upon the 3‐HAA^1^‐adenylate to yield the ester (not the amide; Figure 3 c).

To probe the amide vs. ester hypothesis, NatL2 was incubated with a range of substrate analogues, 2‐amino benzoic acid, 3‐amino benzoic acid, 2,3‐diamino benzoic acid, SA, 3‐hydroxybenzoic acid, and 2,3‐dihydroxybenzoic acid, in the presence of Mg^2+^ and ATP. No reaction was observed with 2‐amino benzoic acid, 3‐amino benzoic acid, and 2,3‐diamino benzoic acid. The failure of these molecules, which all have amine groups, to react argues that NatL2 does not catalyze amide bond formation. In contrast, 3‐hydroxybenzoic acid and 2,3‐dihydroxybenzoic acid both reacted in the presence of NatL2 to yield the esters 3‐((3‐hydroxybenzoyl)oxy)benzoic acid (3‐HBA_2_, **4**) and 3‐((2,3‐dihydroxybenzoyl)oxy)‐2‐hydroxybenzoic acid (**5**) (Figure 3 d and Supporting Information, Figure S1). The identity of **4** was confirmed by NMR spectroscopy (Supporting Information, Figure S8). SA, which lacks a hydroxyl group at the *meta* position, gives no reaction, indicating that the hydroxyl must be at the *meta* position for reaction. The formation of esters with substrate analogues with a *meta* hydroxyl is evidence that NatL2 catalyzes ester formation with 3‐HAA. We assign compound **1** (Figure 1 f) as this ester (Figure 3 c).

### Structural Biology of NatAM

**Figure 4:**
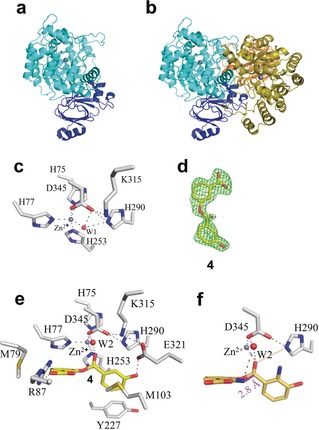
Structural biology of NatAM. a) Overall structure of NatAM reveals a larger domain (cyan) and a smaller domain (dark blue). The zinc ion is shown as a gray sphere. b) NatAM exists as a dimer. The second monomer is colored olive (larger domain) and orange (smaller domain). c) The zinc ion coordination sphere is typical for a hydrolase enzyme. Carbon atoms in the protein are colored gray, other atoms colors are as Figure 2 a. d) Simulated‐annealing Fo‐Fc omit density (2.5 σ) for **4** in NatAM:**4** complex. e) Compound **4** binds at the active site of NatAM where it displaces a water (W1). A second conserved W2 is bound to the ligand and protein. f) In silico modelling of the true substrate into the active site of NatAM. The nitrogen atom is positioned 2.8 Å from the carbonyl (magenta line) ready to attack and form a hemiorthoamide. The hydrogen bond to His290 is shown as an orange dashed line. Carbons are colored gray for protein, yellow for ligands.

The NatAM structure was determined to 1.7 Å resolution using the Zn^2+^ anomalous signal (Figure 4 a). The asymmetric unit contains two monomers of NatAM, which PISA indicates form a homodimer (Figure 4 b). Residues 1–493 in chain A and 2–494 chain B were traced, and the monomers superimpose with an rmsd of 0.238 Å over 486 Cα atoms. NatAM comprises two domains, including a smaller domain (residues 1–70 and 400–454) composed of a β‐roll and two α‐helices. The larger domain (residues 71–399) has a central core reminiscent of a distorted (β/α)_8_ barrel (Figure 4 a). The zinc ion in *apo* NatAM is located in the centre of (β/α)_8_ arrangement where it is coordinated by His75, His77, His253, and one water molecule in an approximate tetrahedral arrangement (Figure 4 c). Asp345 contributes to the coordination sphere but with a zinc–oxygen distance of 2.7 Å, giving a distorted trigonal bipyramid. The water molecule hydrogen bonds to Asp345 and His290. Soaking of *apo* NatAM crystals with AMP, ATP, AMPPNP, compounds **2** or **3** gave no complex.

The structures of NatAM with 3‐HAA (1.25 Å resolution) and 3‐HBA (1.17 Å resolution) were obtained by soaking and show that 3‐HAA and 3‐HBA molecules superimpose (Supporting Information, Figure S9), as do the protein structures, which are largely unchanged from the *apo*. In both complexes, the oxygen atom from the carboxylate coordinates to the zinc ion displacing the water. The other oxygen atom of the carboxylate makes a hydrogen bond to His290, which makes a salt bridge to Asp345. The 3‐hydroxyl group forms a hydrogen bond with Glu321. The 2‐amino group of 3‐HAA bridges the side chains of Asp345 and Glu321. The benzene ring of the acid group sits in a hydrophobic pocket formed by Met103, Phe116, Tyr227, His253, Ala256, and Leu257 (Supporting Information, Figure S9).

We soaked NatAM crystals with a substrate mimic, ester **4** (Figure 4 d) and obtained a 1.8 Å resolution structure. The acid portion (of the ester) of **4** superimposes with the 3‐HAA monomer and makes similar interactions with the protein. The carbonyl oxygen of the ester displaces the water from the zinc. The protein structure is unchanged with one notable exception that the loop (Phe84–Gly88) has adjusted its conformation (Supporting Information, Figure S9) so that Arg87 salt bridges to the carboxylate of the other ring (alcohol portion) of the ester (Figure 4 e). The benzene ring of the alcohol π–π stacks with His77 in a hydrophobic pocket formed by Met79, Phe116, and Phe119. In 3‐HBA_2_, the planes of the two benzene rings are offset by 52° (Figure 4 e). Facile in silico modification of **4** by placing an amine group onto each benzene rings gives a model of the true substrate and positions the 2‐amine of the alcohol portion 2.8 Å from the ester with an angle (N_amine_–C_ester_–O_ester_) of 74°, consistent with attack to form a hemiorthoamide intermediate (Figure 4 f). His290 is positioned to transfer a proton onto the hemiorthoamide oxygen that would form upon attack by the amine. The mutant H290A completely abolished production of **3** (Supporting Information, Figure S10). A water molecule bound to Lys315 and the ester is conserved in the structures. The mutant K315A shows a 10‐fold reduction in activity (Supporting Information, Figure S10).

### Synthetic Utility of NatL2 and NatAM

**Figure 5:**
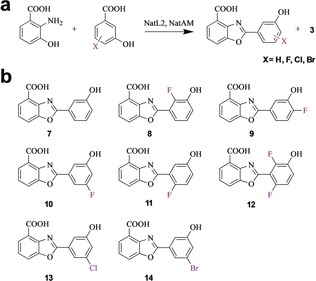
Novel benzoxazoles synthesized by NatL2/NatAM. a) The reaction scheme uses 3‐HAA and derivatives of 3‐HBA in the presence of both NatL2 and NatAM. b) Novel benzoxazole analogues made enzymatically.

Halogenation is a very common medicinal chemistry modification, fluorine especially has a unique chemistry.^49^ Moreover, halogens allow access to Heck coupling chemistry, thus facile production of halogenated benzoxazoles is desirable. Guided by our structures of NatL2 and NatAM, we were able to make benzoxazoles with fluoro substituents at positions, C2, C4, and C5, as well as chloro and bromo substitutions at C5 within the ring adenylated by NatL2 (Figure 5 and Supporting Information, Figure S13). We were able to detect a product corresponding to a fluoro substituent at C6 and we observed a trace of the difluoro analogue **12** (the fluorine atoms may deactivate the carboxylate for adenylation). The system was able to process analogues with chloro and bromo substituents at C5. These halogens would either require significant movement of the Tyr227 in NatAM or alternatively that the phenyl ring rotates by 180° around its C1–C4 axis such that that the bromo and hydroxyl substituents swap binding sites. That the system tolerates a wide range of substrates shows it holds promise as a synthetic tool.

## Conclusion

The biosynthesis of benzoxazoles in contrast to (thi)oxazole biosynthesis has remained uncharacterized, an important oversight given its prevalence in medicines and natural products. We reconstituted the biosynthesis of the benzoxazole moiety found in nataxazole by adding two enzymes (NatL2, NatAM), to 3‐HAA, ATP, and Mg^2+^. NatL2 belongs to the PaaK family, a subset of acyl‐CoA synthetases that operate via acyl adenylates. To enable CoA binding and thus thiol attack on the acyl‐AMP intermediate, PaaK enzymes undergo a major conformational change where the C‐terminal domain rotates to make an “open” form. This motion is prevented in NatL2 by a C‐terminal extension that locks the structure in the “closed” form (Supporting Information, Figure S5). FAALs are locked in the “closed” arrangement and unable to catalyze CoA thioester formation due to an insertion into the hinge between the C‐ and N‐terminal domain.^41^, ^42^, ^43^ FAALs and NatL2 appear to have diverged from the PaaK archetype but each has evolved a different strategy to lock the dimeric arrangement in a closed form to prevent CoA attack on the acyl‐AMP.

Previous studies had predicted that BomJ, (NatL2 homologue) synthesized an amide linkage between two 3‐HAA monomers via an acyl‐AMP intermediate.^38^ Our data establish that the amide **2** is not the true product of NatL2, rather NatL2 catalyzes an ester linkage between two 3‐HAA molecules, compound **1**. We conclude that the previous report^38^ of an amide intermediate is incorrect, rather the amide is an isobaric shunt product. Addition of **2** to cells with a deletion mutant (Δ*bomO*) in the 3‐HAA pathway was previously shown to result in a small amount of benzoxazole.^38^ We suggest this result^38^ is explained by the breakdown of **2** to 3‐HAA that is then processed to the product.

**Figure 6:**
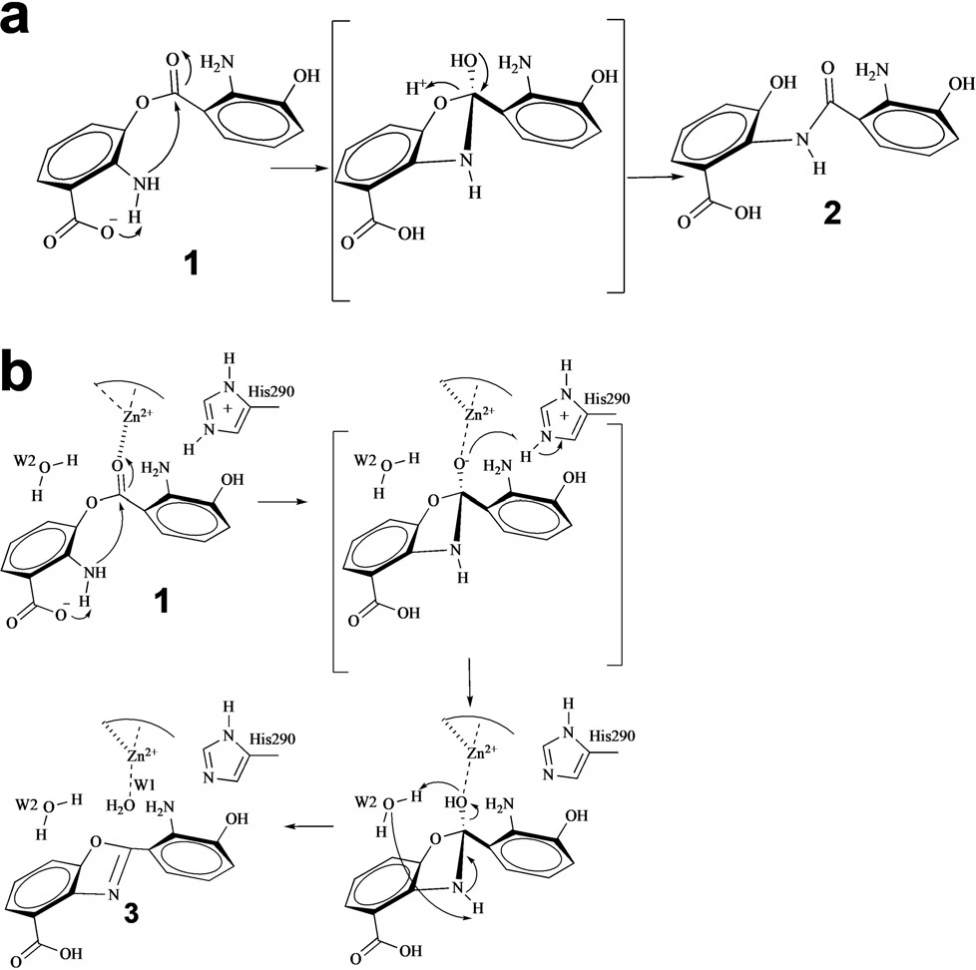
Proposed catalytic mechanism for NatAM. a) Spontaneous conversion of ester intermediate **1** into amide **2** in aqueous solution without NatAM. b) Catalytic mechanism for cyclization and dehydration via the hemiorthoamide intermediate (in brackets) in the presence of NatAM. The hemiorthoamide intermediate is stabilized by proton transfer from His290.

Our data show that **1** spontaneously converts to **2** so rapidly it prevented isolation of **1** in our hands explaining the isolation of **2** previously.^38^ The spontaneous conversion of **1** is predicted to occur via a tetrahedral hemiorthoamide intermediate formed by attack of the amine on the ester (Figure 6 a). In RiPP^24^ and NRP^29^, ^30^, ^31^, ^32^ systems, oxazoline biosynthesis also proceeds via this hemiorthoamide intermediate (phosphorylated in RiPP) but it is formed by the attack of hydroxyl upon an amide (Figure 1 b).

NatAM, belongs to the amidohydrolase family that is known to hydrolyse C−O, P−O, P−S, C−N, C−S, and C−Cl bonds. These enzymes activate water by zinc coordination creating a nucleophilic hydroxide^50^ that then attacks the electrophilic carbon. Where the carbon is sp^2^ hybridized, an addition–elimination reactions occur via a tetrahedral intermediate, for example in adenosine deaminase.^51^, ^52^ The structure of NatAM identifies a water molecule bound to the zinc (Figure 4 c), but this water is displaced in every complex structure and thus seems unlikely to play a hydrolytic role. A complex with **4,** a substrate mimic (Figure 4 e), shows that the amine on the benzene ring is arranged to attack the ester bond (Figure 4 f) and thus form the hemiorthoamide intermediate (Figure 6 a,b). The attack is facilitated by the substrate's carboxylate, which is positioned to abstract the proton from the nitrogen atom. Upon becoming tetrahedral, the oxygen of the ester will move towards the zinc, which would stabilize the negative charge on the oxygen. In solution, the collapse of the hemiorthoamide intermediate occurs by elimination of the alcohol to yield the amide. NatAM however, catalyzes the elimination of water from the hemiorthoamide to form the benzoxazole. Simple atom counting requires that two protons are removed from the substrate amine (which attacks the ester bond) and two protons are added to the oxygen of ester (which eliminates as water) during the reaction. The structure discloses two plausible candidates for proton donation to the ester oxygen, His290 and a conserved water molecule that is hydrogen bonded to both the ester and to Lys315. Mutation of His290 eliminates activity but mutation of Lys315 reduces catalytic activity. We propose that one proton is supplied by His290 and the other from the conserved water (Figure 6 b). We note the amine is positioned for intramolecular proton transfer to the substrate's carboxylic acid (Figure 6 b). In this mechanism, the conserved water molecule forms a six‐membered ring as it transfers a proton from the nitrogen to the oxygen atom (Figure 6 b). This is the third catalytic strategy for forming and controlling the fate of a hemiorthoamide intermediate and is an elegant example of the interplay between chemistry and evolution.^24^, ^53^, ^54^

The nataxazole system reveals a novel series of reactions involving an unstable ester that forms a hemiorthoamide that is dehydrated by an amidohydrolase. Both enzymes belong to well‐known superfamilies but catalyze new reactions. These data provide a basis for a two‐enzyme system that can produce novel benzoxazoles.

## Conflict of interest

The authors declare no conflict of interest.

## Supporting information

As a service to our authors and readers, this journal provides supporting information supplied by the authors. Such materials are peer reviewed and may be re‐organized for online delivery, but are not copy‐edited or typeset. Technical support issues arising from supporting information (other than missing files) should be addressed to the authors.

Supplementary
