# Supplementary material for: The Biosynthesis of the Benzoxazole in Nataxazole Proceeds via an Unstable Ester and has Synthetic Utility
Source: Angew Chem Int Ed Engl. 2020 Feb 11;59(15):6054–61. doi: 10.1002/anie.201915685 (PMC7204872; doi:10.1002/anie.201915685)
Supplement: Supplementary file 1 — Supplementary [file ANIE-59-6054-s001.pdf]

Angewandte Chemie
Eine Zeitschrift der Gesellschaft Deutscher Chemiker

Supporting Information

The Biosynthesis of the Benzoxazole in Nataxazole Proceeds via an Unstable Ester and has Synthetic Utility

Haigang Song, Cong Rao, Zixin Deng, Yi Yu, and James H. Naismith**

anie_201915685_sm_miscellaneous_information.pdf

Author Contributions

All authors contributed to the writing of the manuscript and analysis of the data. J.H.N. and Y.Y. initiated the project; J.H.N., Y.Y., and H.S. designed the experiments, interpreted data and wrote the manuscript; J.H.N. and H.S. carried out the structural biology; C.R., Z.D., and Y.Y. performed purification and in vitro assays, characterization of isolated the compounds using NMR and MS; C.R., Z.D., and Y.Y. designed the primers and performed the mutagenesis study. All authors read and approved the final manuscript.

Table of Contents

1. Experimental Procedures 3
2. Characterization of compounds 5
3. Tables 6
4. Supporting Figures 10
References 28

1. Experimental Procedures

Buffers, salts, 3-HAA and other chemicals were purchased from Sigma-Aldrich. Crystallization screens were bought from Hampton Research, or Molecular Dimensions. Gilson assembly kits were purchased from YEASEN. PPi detection kit was purchased from Invitrogen.

Protein expression and purification of NatL2
The nucleotide sequence of *natL2* (GenBank: LN713864.1) was synthesized by GENEWIZ (Suzhou, China) and cloned into pET-28a(+) vector (Novagen) using Gilson assembly methods to yield pWDY1211. For protein expression, pWDY1211 was transformed into *E. coli* BL21 (DE3) cells. Cells were grown to OD_{600} 0.6~0.8 at 37 °C in LB broth medium with 50 μg/L kanamycin before being cooled to 18 °C. Protein expression ws induced by addition of 0.2 mM IPTG. The cells were then harvested after 20 h incubation and resuspended in lysis buffer containing 25 mM Tris (pH 8.0), 0.5 M NaCl, 10% glycerol, 25 μg/ml DNase and EDTA-free cocktail protein inhibitors (Roche) for 1h in cold room, and lysed by being passed through a cell disruptor (Constant Systems Ltd) at 30 kpsi, twice. The cell lysate was cleared by centrifugation at 40,000 g for 30 min, and filtered through a membrane (0.45 μm). The supernatant was loaded onto a 5 ml HisTrap FF column (GE Healthcare) pre-equilibrated in lysis buffer, washed with lysis buffer supplemented with 20 mM imidazole, and protein was eluted with 250 mM imidazole. Fractions corresponding to NatL2 was then quickly desalted into 25 mM tris buffer containing 10% glycerol, and 0.15 M NaCl. Concentrated fractions of NatL2 was further purified using a size exclusion column (Superdex S200 from GE Healthcare). Well folded NatL2 fractions were collected, concentrated, flash frozen, and stored at -80 °C in buffer containing 25 mM Tris pH 8.0, 10% glycerol and 0.1 M NaCl.

Protein expression and purification of NatAM
The nucleotide sequence of gene *natAM* (from GenBank: LN713864.1) was synthesized by GENEWIZ (Suzhou, China) and cloned into the expression vector pWDYHS01[1] using Gilson assembly methods to yield pWDY1232. The expression vector pWDYHS01 was constructed in our previous study, which harbors an N-His_6-tag originated from pET28a and a PtipA promoter controlled multi-cloning site originated from pGM1190. The plasmid pWDY1232 was then transferred into *Streptomyces albus* by conjugation. The correct transconjugant verified by PCR and sequencing was grown in TSBY medium and then scaled up in YEME medium supplemented with 50 μg/L apramycin. Cells were grown at 28 °C for 48 h, and then induced expression with 50 μg/L thiostrepton for another 3 days. The cells were harvested and resuspended in 40 mL Tris buffer (20 mM Tris·HCl, 300 mM NaCl, pH 8.0). The following purification procedure of NatAM is same as that of NatL2 described above.

Enzymatic assays of wild-type and mutant NatL2 and NatAM
Enzymatic assays were carried out on a 100 μL scale with 2 μM purified NatL2 or NatAM or both, 1 mM ATP, and 1 mM substrate in 50 mM Tris-HCl (pH 8.0). The mixture was incubated at 30 °C for 180 min. The reactions were quenched by adding equal volumes of methanol. The detection of the product was analysed by HPLC, which was carried out on a DIONEX system equipped with P680 HPLC pump, using a DIKMA Diamonsil C18 column (5 μm, 250×4.6 mm) eluted with a linear gradient from 40% B to 85% B for 5–20 min, at a flow rate of 0.8 ml/min (solvent A was 0.1% HCOOH in H_2O and solvent B was 0.1% HCOOH in CH_3CN). The UV monitor was set at 254 nm. The identities of the reaction products were characterized by high resolution MS analysis, which consists of a full scan in positive mode followed by a data dependent fragmentation scan, through a LTQ XL Orbitrap mass spectrometer (Thermo Fisher Scientific Inc.). The following parameters were set: 45V capillary voltage, 250 °C capillary temperature, auxiliary gas flow rate 5 arbitrary units, sheath gas flow rate 30 arbitrary units, 3.5 kV spray voltage, and 100–1500 Amu mass range.

For detection of compound **1**, NatL2 at a concentration of 2 μM was incubated with 1 mM 3-HAA, 1 mM ATP, 10 mM $MgCl_2$, and 50 mM Tris-HCl (pH 8.0) in a volume of 50 μL reaction system for 15 min at 30 °C. Reactions were filtered through a 10 kDa membrane to remove the protein by centrifugation at 4 °C. The filtrate was then subjected to LC-MS analysis as above. To study the conversion of **1** to **2**, the filtrate was further incubated at room temperature for 1 h or 2 h before loaded on LC-MS. To study the conversion of **1** to **3**, the filtrate was incubated with 2 μM NatAM at room temperature for 1h before loading onto LC-MS.

Assays of NatL2
NatL2 and NatAM were incubated with 5 mM EDTA at 4 °C overnight. The enzyme activity was then tested in a volume of 100 μL consisting of 2 μM EDTA treated proteins, 1 mM ATP, and 1 mM 3-HAA in 50 mM Tris-HCl (pH 8.0) but no activity was detected. 10 mM $MgCl_2$, and 10 mM $ZnCl_2$ were added to the reaction and product observed.

Structural characterization of compound 2, 3 and 4
For structural characterization of compound **2**, **3** and **4** generated in NatL2 and NatAM reactions, a 100 mL aqueous solution containing 50 mM Tris-HCl pH 8.0, 2 μM purified enzyme(s), 10 mM $MgCl_2$, 1 mM ATP and 1 mM substrate was prepared. All assays were mixed with a pipette and divided into 1 mL per tube, incubated at 30 °C for 3 h and finally quenched by adding equal volumes of methanol. Proteins were removed by centrifugation. The targeted compounds were enriched and isolated with macro-porous adsorptive resins and further purified by HPLC semi-preparation using parameters described above. The purified compounds were concentrated and

resolved in appropriate solvent and characterized using NMR. The NMR data of compound **2** and **3** were recorded on the Bruker Avance 500 MHz NMR spectrometry and the NMR data of compound 4 were recorded on a Bruker Avance 400 MHz NMR spectrometry.

Site-directed mutagenesis of NatL2 and NatAM
The whole plasmid pWDY1211 or pWDY1232 was amplified by high-fidelity PCR using each primer pair. After checking the PCR products by agarose gel electrophoresis, 2 µL of the PCR product was treated with the restriction enzyme Dpn I , and the total 20 µL reaction solution was incubated at 37 °C for 1.5 h. 2.5 µL of the resulting solution was used for transformation of *E. coli* DH10B competent cells. The positive clones were picked and verified by DNA sequencing.

Substrate scope of NatL2 and NatAM
Various halogenated derivatives of 3-HBA were incubated with NatL2 and NatAM in the presence of 3-HAA. Typically, the reactions were carried out on a 100 µL scale with 2 µM purified NatL2/NatAM, 1 mM ATP, 1 mM 3-HAA, 1 mM halogenated derivative and 10 mM $MgCl_2$ in 50 mM Tris-HCl (pH 8.0). The mixture was incubated at 30 °C for 180 min. Benzoxazoles and amide shunt products were analysed by LC-MS and confirmed by high resolution MS.

Crystallization, data collection and structure determination
Freshly prepared NatL2 was initially screened as *apo* form and crystals were obtained at 4 ºC. These crystals diffracted poorly at the Diamond synchrotron and the structure was not solved. We incubated with 5 mM ATP and 5 mM $MgCl_2$ for 1 h before setting up crystallization. Plate shaped crystals of NatL2 were successfully obtained within 2 weeks using hanging drop diffusion methods by mixing the 1 µl of protein (40 mg/ml of protein, 5 mM ATP and 5 mM magnesium chloride) with 1 µl of reservoir solution (0.2 M KSCN, 0.1 M sodium citrate pH 6.0, 30% PEG MME 2K) at room temperature. Crystals were fished, transferred to cryo-protectant (0.2 M KSCN, 0.1 M sodium citrate pH 6.0, 40% PEG MME 2000) and then flash-frozen by plunge into liquid nitrogen.

Crystals grown in the presence of ATP were soaked overnight in mother liquor supplemented with either with 20 mM AMPPNP, 20 mM ATP, saturating concentrations of **4**, saturating concentration of 3-HAA or combined saturating 3-HAA and 20 mM AMPPNP. Co-crystallization of NatL2 and 5 mM 3-HAA, 5 mM AMP and 5 mM $MgCl_2$, was also performed but gave no new information. To obtain NatL2:SA complex structure, co-crystallized NatL2:3-HAA crystals were soaked in 300 mM salicylic acid overnight. Crystals were then transferred into similar cryo-protectant supplemented with varied ligands before being flash-frozen in liquid nitrogen. X-ray fluorescence scan spectrum (MCA) suggests existence of zinc ion in the crystal and phase information was obtained from the zinc anomalous signal using MAD methods at peak (1.2828 Å), inflection (1.2832 Å) and high remote wavelength (1.2697 Å) (**Table S3 and S4**).

NatAM at a concentration of 40-50 mg/ml was screened for crystallisation. Crystals were obtained in two conditions after two weeks at 4 ºC. Crystals belonging to space group C121 (*C*2) are grown in the condition containing 0.05 M $MgCl_2$, 0.1 M HEPES pH 7.5 and 30% v/v polyethylene glycol monomethyl ether 550, while crystals belongs to P $2_12_12_1$ or $P2_12_12$ were grown in the condition containing 0.2 M $MgCl_2$, 0.1 M Bis-Tris pH 6.5 and 25% w/v polyethylene glycol 3,350. Full size crystals were later transferred in the mother liquor supplied with 40-45% polyethylene glycol 3,350. NatAM crystals were soaked in its mother liquor containing 20 mM zinc chloride for several minutes, and then flash-frozen in liquid nitrogen with 40% PEG 3350 as cryo-protectant.

To obtain complex structures of NatAM, apo NatAM crystals were soaked overnight in the mother liquor supplied with 20 mM AMP, ATP, AMPPNP, 3-HAA, 3-HBA, **2** and **3** at saturated concentration before being flash-frozen in similar cryoprotectant. NatAM crystals were also soaked with **4** at saturated concentration from 2 hours to one week. Higher occupancy of **4** was observed by using longer soaking times.

All the X-ray diffraction data were recorded at Diamond Light Source beamlines (I03, I04, I04-1, and I24) which we gratefully acknowledge. The diffraction images were reduced, integrated, and scaled using xia2[2], DIALS[3] or autoPROC[4]. To obtain phase information, the NatL2:AMP crystals were also grown in the presence of 5 mM manganese (II) chloride, however X-ray fluorescence scan spectrum suggests a much higher abundance for zinc instead of manganese ion. A fluorescence scan was then used to locate the Zn *K* edge as 1.2828 Å at I04. Multiple-wavelength data were then collected at wavelengths of 1.2828 Å (peak), 1.2832 Å (inflection) and 1.2697 Å (high energy remote). For NatAM crystals, X-ray fluorescence scan spectrum indicates strong signal of zinc and MAD data were collected at wavelengths of 1.2821 Å (peak), 1.2826 Å (inflection) and 1.2626 Å (high energy remote). AutoSHARP[5] was used to locate the two zinc atom sites in the asymmetric unit and initial model was obtained by using autobuilding programme ARP/wARP[6] or Phenix AutoBuild wizard[7]. The initial model was further refined using Refmac[8], Phenix[9], manually built with Coot[10], and improved using PDB-redo[11]. All the other structures were solved by molecular replacement using PHASER[12] with NatL2-AMP complex structure or *apo* NatAM structure as the initial models and refined as above.

All crystallographic figures were generated using Pymol (Schrödinger LLC) or CCP4mg[13]. All the coordinates and structural factors files were deposited to PDB with accession code 6SIW, 6SIX, 6SIY, 6SIZ, 6SJ0, 6SJ1, 6SJ2, 6SJ3, and 6SJ4. Secondary structure of both NatL2 and NatAM were assigned using DSSP web server[14]. Structure based sequence alignment was created using MUSCLE[15] or ClustalX[16] and ESPript[17].

2. Characterisation of compounds

Compound 2: **^{1}H-NMR**, δ_H (500 MHz, DMSO-*d*6): 7.10 (1H, dd, *J* = 8.03, 1.34 Hz, H-4), 7.16 (1H, t, *J* = 7.84 Hz, H-5), 7.35 (1H, dd, *J* = 7.62, 1.25 Hz, H-6), 6.81 (1H, m, H-11), 6.46 (1H, t, *J* = 7.87 Hz, H-12), 7.27 (1H, m, H-13), 9.74(1H, s , OH-3), 9.59(1H, s, OH-10), 10.13(1H, s, NH-14).
^{13}C-NMR, δ_C (125 MHz, DMSO-*d*6): 126.10 (C, C-1), 127.67 (C, C-2), 151.64 (C, C-3), 120.59 (CH, C-4), 125.83 (CH, C-5), 121.20 (CH, C-6), 168.53 (C, C-7), 114.18 (C, C-8), 139.59 (C, C-9), 144.80 (C, C-10), 115.86 (CH, C-11), 114.48 (CH, C-12), 119.17 (CH, C-13), 168.30 (C, C-14).

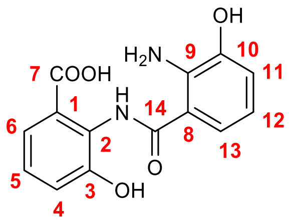

Compound 3: **^{1}H-NMR**, δ_H (500 MHz, MeOD-*d*4): 7.83 (1H, d, *J* = 6.48 Hz, H-4), 7.42 (1H, t, *J* = 7.85 Hz, H-5), 7.97 (1H, d, *J* = 7.61 Hz, H-6), 6.84 (1H, d, *J* = 7.32 Hz, H-11), 6.59 (1H, t, *J* = 7.38 Hz, H-12), 7.54 (1H, d, *J* = 7.80 Hz, H-13).
^{13}C-NMR, δ_C (125 MHz, MeOD-*d*4): 123.09 (C, C-1), 142.91 (C, C-2), 151.24 (C, C-3), 115.33 (CH, C-4), 125.07 (CH, C-5), 127.75 (CH, C-6), 168.90 (C, C-7), 108.63 (C, C-8), 140.22 (C, C-9), 146.20 (C, C-10), 117.09 (CH, C-11), 116.98 (CH, C-12), 119.99 (CH, C-13), 166.28 (C, C-14).

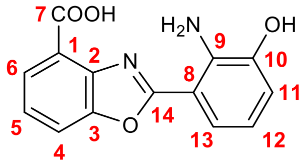

Compound 4: **^{1}H-NMR**, δ_H (400 MHz, MeOD-*d*4): 7.83 (1H, s, H-2), 7.66 (1H, d, *J* = 7.70 Hz, H-4), 7.56 (1H, overlapped, H-5), 7.96 (1H, d, *J* = 7.71 Hz, H-6), 7.58 (1H, overlapped, H-9), 7.11 (1H, dd, *J* = 8.10, 2.55 Hz, H-11), 7.38 (1H, t, *J* = 7.38 Hz, H-12), 7.45 (1H, dd, *J* = 8.12, 2.12 Hz, H-13).
^{13}C-NMR, δ_C (100 MHz, MeOD-*d*4): 133.74 (C, C-1), 124.01 (CH, C-2), 152.44 (C, C-3), 127.48 (CH, C-4), 130.67 (CH, C-5), 128.19 (CH, C-6), 168.91 (C, C-7), 131.61 (C, C-8), 117.47 (CH, C-9), 159.04 (C, C-10), 122.12 (CH, C-11), 130.90 (CH, C-12), 122.19 (CH, C-13), 166.45 (C,C-14).

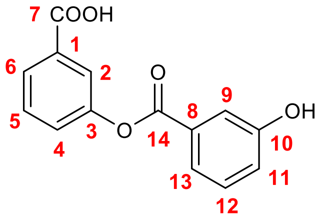

3. Tables

Plasmids/strains	Relevant genotype/comments
**Plasmids**	
pET-28a(+)	Expression vector of NatL2
pWDYHS01	His_6-tag was inserted downstream of tipA promoter in protein overexpression vector in Streptomyces (tsr, aphII, PtipA promoter, RBS, N/C-terminal His6-tag, fd terminator)
pWDY1211	NatL2 overexpression construction
pWDY1222	Point mutation of K418A in NatL2 construction
pWDY1226	Point mutation of R156A in NatL2 construction
pWDY1228	Point mutation of R156K in NatL2 construction
pWDY1232	NatAM overexpression construction
pWDY1233	Point mutation of H290A in NatAM construction
pWDY1234	Point mutation of K315A in NatAM construction
pWDY1235	Point mutation of D345A in NatAM construction
**strains**	
E.coli	
WDY1211	NatL2 overexpression strain
WDY1222	K418A mutant of NatL2 overexpression strain
WDY1226	R156A mutant of NatL2 overexpression strain
WDY1228	R156K mutant of NatL2 overexpression strain
Streptomyces	
WDY1232	NatAM overexpression strain
WDY1233	H290A mutant of NatAM overexpression strain
WDY1234	K315A mutant of NatAM overexpression strain
WDY1235	D345A mutant of NatAM overexpression strain

Table S1. Plasmids and strains used in this study.

Table S2. Primers used in NatL2 and NatAM mutagenesis.

Primer Name	Sequence (5'-3')
NatL2_K418A_F	CAGCGCGACATCCTC**GCC**GCCCGCTACCTCTTC
NatL2_K418A_R	GAAGAGGTAGCGGGC**GGC**GAGGATGTCGCGCTG
NatL2_R156A_F	GTCCCCGGCGACGCC**GCC**TCGCTGGCCACCCCG
NatL2_R156A_R	CGGGGTGGCCAGCGA**GGC**GGCGTCGCCGGGGAC
NatL2_R156K_F	GTCCCCGGCGACGCC**AAG**TCGCTGGCCACCCCG
NatL2_R156K_R	CGGGGTGGCCAGCGA**CTT**GGCGTCGCCGGGGAC
NatAM_H290A_F	CGGCTGATGGCCGGG**GCC**AGCGCCTTCCTGGAC
NatAM_H290A_R	GTCCAGGAAGGCGCT**GGC**CCCGGCCATCAGCCG
NatAM_K315A_F	CCCACTCCCCCGGC**GCC**TACGGCCCGTCGGG
NatAM_K315A_R	CCCGACGGGCCGTA**GGC**GCCGGGGGAGTGGG
NatAM_D345A_F	GTCGCTGTCGACC**GCC**GCGGCGGCGCTGCC
NatAM_D345A_R	GGCAGCGCCGCCGC**GGC**GGTCGACAGCGAC

Table S3. Data collection, phasing and refinement statistics for NatL2.

	NatL2-AMP			NatL2:AMP (Native)	NatL2: AMPPNP	NatL2:AMP: 3-HAA	NatL2:AMPPNP: 3-HAA	NatL2:SA
PDB Entry				**6SIW**	**6SIX**	**6SIY**	**6SIZ**	**6TM4**
**Data collection**								
Space group	C 2 2 2_1	C 2 2 2_1	C 2 2 2_1	C 2 2 2_1	C 2 2 2_1	C 2 2 2_1	C 2 2 2_1	C 2 2 2_1
Cell dimensions								
a, *b*, *c* (Å)	107.0, 138.3, 129.9	107.1, 138.4, 130.1	107.1, 138.4, 130.1	107.1, 138.1, 130.4	107.4, 138.1, 130.5	107.4, 138.6, 130.4	107.5, 138.8, 130.7	107.5, 139.2, 129.7
α, β, γ (°)	90, 90, 90	90, 90, 90	90, 90, 90	90, 90, 90	90, 90, 90	90, 90, 90	90, 90, 90	90, 90, 90
	Peak	***Inflection***	***Remote***					
Wavelength (Å)	1.2828	1.2832	1.2697					
Resolution (Å)	61.03-2.33 (2.37-2.33)	69.18-2.41 (2.45-2.41)	61.08-2.49 (2.53-2.49)	84.65-1.96 (1.99-1.96)	130.52-1.88 (1.91-1.88)	69.29-1.95 (1.98-1.95)	51.80-1.77 (1.80-1.77)	71.13-1.89 (1.94-1.89)
R_{merge}	0.161 (0.967)	0.150 (0.950)	0.156 (0.939)	0.108 (0.901)	0.081 (1.810)	0.060 (0.841)	0.101 (1.779)	0.119 (1.628)
$I / \sigma I$	9.4 (2.1)	10.8 (2.2)	10.6 (2.2)	12.8 (1.9)	12.0 (0.9)	13.2 (1.4)	11.4 (1.1)	9.4 (1.3)
Completeness (%)	99.9 (97.5)	100.0 (97.7)	100.0 (98.8)	100.0 (98.8)	99.8 (95.9)	100.0(99.6)	99.5 (99.0)	100.0 (100.0)
Redundancy	12.9 (12.9)	12.9 (13.2)	12.9 (13.0)	10.7 (10.0)	7.3 (6.7)	7.1 (6.5)	7.9 (8.3)	6.5 (6.7)
No. unique reflections	41467 (2023)	37621 (1812)	34184 (1667)	69487 (3406)	78902 (3759)	70886 (3449)	94479 (4655)	77807 (5703)
$CC_{1/2}$	0.999 (0.504)	0.999 (0.604)	0.999 (0.593)	0.999 (0.522)	0.998 (0.465)	0.999 (0.510)	0.994 (0.557)	0.997 (0.676)
**Refinement**								
Resolution (Å)				(84.65-1.96) (1.99-1.96)	130.52-1.88 (1.91-1.88)	69.29-1.95 (1.98-1.95)	51.80-1.77 (1.80-1.77)	
R_{work} / R_{free}				0.197/0.234	0.181/0.210	0.186/0.223	0.200/0.229	0.1974/0.2305
No. atoms								
Protein				6858	6894	6813	6861	6727
Ligand/ion				71	108	129	125	67
Water				261	390	169	221	160
B-factors (Å^2)								
Protein				44.2	46.1	63.7	52.5	50.6
Ligand/ion				58.3	52.4	71.2	50.4	48.2
Water				40.5	45.0	52.9	46.7	38.1
R.m.s deviations								
Bond lengths (Å)				0.007	0.007	0.007	0.006	0.009
Bond angles (°)				1.416	1.463	1.455	1.398	1.551
Ramachandran								
Allowed (%)				99.65	100	99.77	99.77	99.88
Outliers (%)				0.35	0	0.23	0.23	0.12

*Values in parentheses are for highest-resolution shell.

SUPPORTING INFORMATION

Table S4. Data collection, phasing and refinement statistics for NatAM.

	NatAM			*Apo* NatAM (Native)	*Apo* NatAM (form II)	NatAM:3-HAA	NatAM:3-HBA	NatAM:**4**
				**6SJ0**	**6SJ1**	**6SJ2**	**6SJ3**	**6SJ4**
**Data collection**								
Space group	P $2_1 2_1 2_1$	P $2_1 2_1 2_1$	P $2_1 2_1 2_1$	P $2_1 2_1 2_1$	C 2	P $2_1 2_1$ 2	P $2_1 2_1$ 2	C 2
Cell dimensions								
a, *b*, *c* (Å)	81.9, 82.3, 141.4	81.9, 82.3, 141.4	81.9, 82.3, 141.5	81.8, 82.8, 141.6	135.6, 54.5, 141.5	99.8, 118.9, 83.3	99.3, 117.3, 83.0	135.3, 54.3, 139.8
α, β, γ (°)	90, 90, 90	90, 90, 90	90, 90, 90	90, 90, 90	90, 111.87, 90	90, 90, 90	90, 90, 90	90, 111.45, 90
	Peak	***Inflection***	***Remote***					
Wavelength (Å)	1.2821	1.2826	1.2626					
Resolution (Å)	71.10-1.75 (1.78-1.75)	53.71-1.76 (1.79-1.76)	53.65-1.78 (1.81-1.78)	53.81-1.75 (1.78-1.75)	67.36-2.06 (2.10-2.06)	83.32-1.25 (1.28-1.25)	117.39-1.17 (1.20-1.17)	65.08-1.81 (1.86-1.81)
R_{merge}	0.211 (2.656)	0.209 (2.644)	0.217 (2.686)	0.155 (1.648)	0.178 (1.370)	0.078 (1.574)	0.105 (1.131)	0.059 (1.087)
$I / \sigma I$	14.4 (1.1)	14.3 (1.1)	13.9 (1.1)	12.1 (1.7)	11.6 (2.1)	12.3 (1.0)	8.1 (1.1)	10.5 (1.1)
Completeness (%)	99.9 (99.1)	99.9 (99.1)	99.9 (99.7)	100 (100)	99.5 (96.5)	97.9 (95.0)	94.4 (64.7)	100 (100)
Redundancy	29.3 (28.0)	29.3 (27.8)	29.4 (29.1)	10.5 (10.9)	12.4 (10.3)	7.0 (5.5)	6.6 (3.9)	3.8 (3.7)
No. unique reflections	96684 (4733)	95180 (4571)	92151 (4523)	97478 (4828)	59518 (2895)	266362 (18918)	306139 (15287)	86621 (6355)
$CC_{1/2}$	0.999 (0.575)	0.999 (0.563)	0.999 (0.562)	0.997 (0.471)	0.997 (0.494)	0.999 (0.480)	0.996 (0.508)	0.997 (0.587)
**Refinement**								
Resolution (Å)				53.81-1.75 (1.78-1.75)	67.36-2.06 (2.10-2.06)	83.32-1.25 (1.28-1.25)	117.39-1.17 (1.20-1.17)	65.08-1.81 (1.86-1.81)
R_{work} / R_{free}				0.181/0.209	0.182/0.219	0.164/0.178	0.136/0.156	0.184/0.210
No. atoms								
Protein				7498	7477	7919	7690	7527
Ligand/ion				43	2	31	50	61
Water				372	236	925	992	295
B-factors (Å^2)								
Protein				30.0	32.7	16.7	15.5	38.4
Ligand/ion				65.9	28.7	21.4	27.8	53.8
Water				31.9	28.6	26.4	28.1	34.9
R.m.s deviations								
Bond lengths (Å)				0.006	0.006	0.007	0.008	0.009
Bond angles (°)				1.402	1.399	1.440	1.498	1.553
Ramachandran								
Allowed (%)				99.8	99.8	99.8	100	99.8
Outliers (%)				0.2	0.2	0.2	0	0.2

*Values in parentheses are for highest-resolution shell.

Table S5. B-factors for SA^2 in the NatL2:SA complex structure.

Atom (SA^2)	B-factor (Å^2)
O2'	45.3
C1'	44.9
O1'	42.6
C1	46.5
C6	46.7
C5	50.5
C4	51.6
C3	52.4
C2	49.7
O2	51.8

4. Supporting Figures

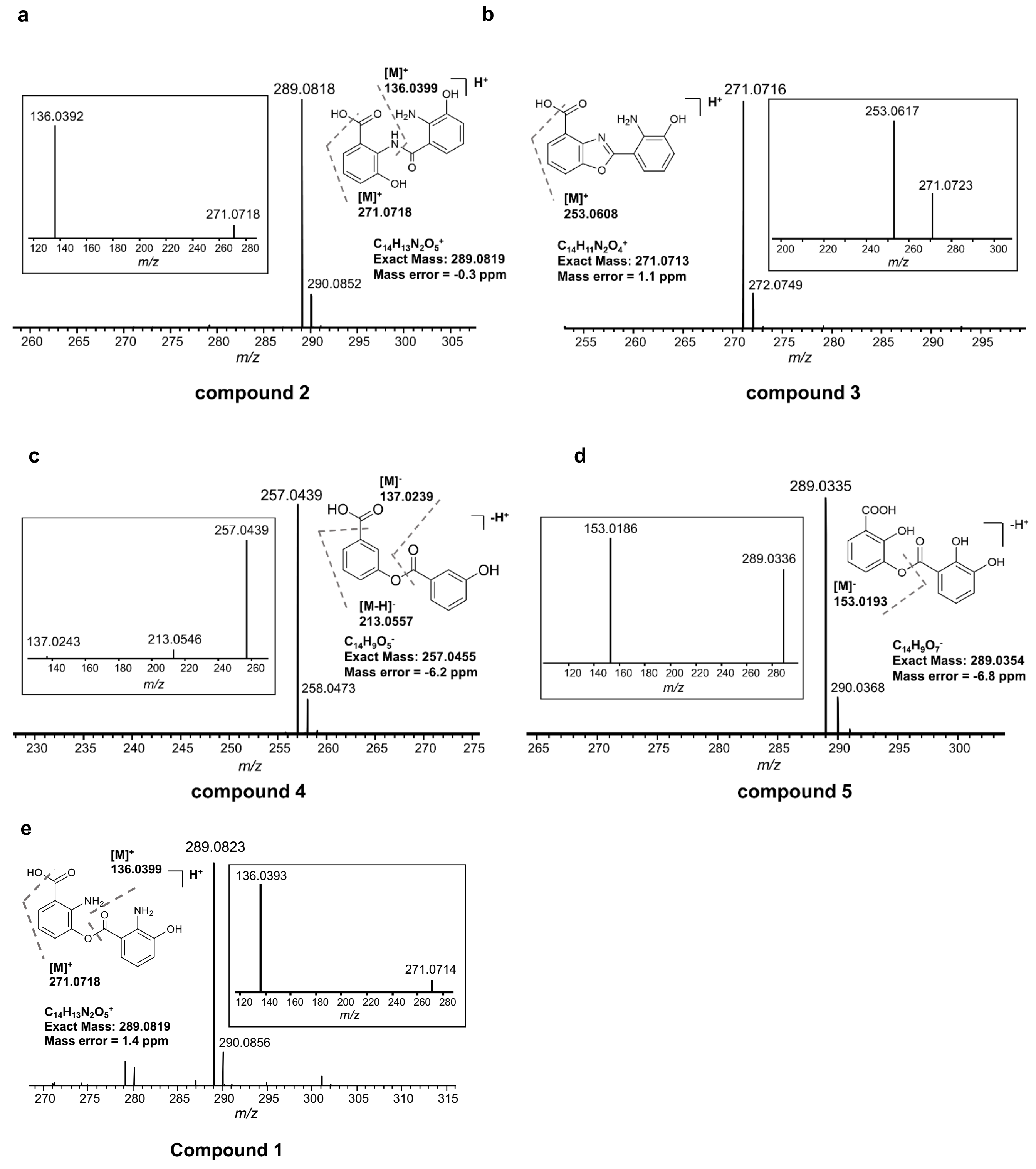

Figure S1. MS spectrum and MS/MS analysis of compounds **2** (**a**), **3** (**b**), **4** (**c**), **5** (**d**) and **1** (**e**). Inset plots show the MS/MS analysis.

a

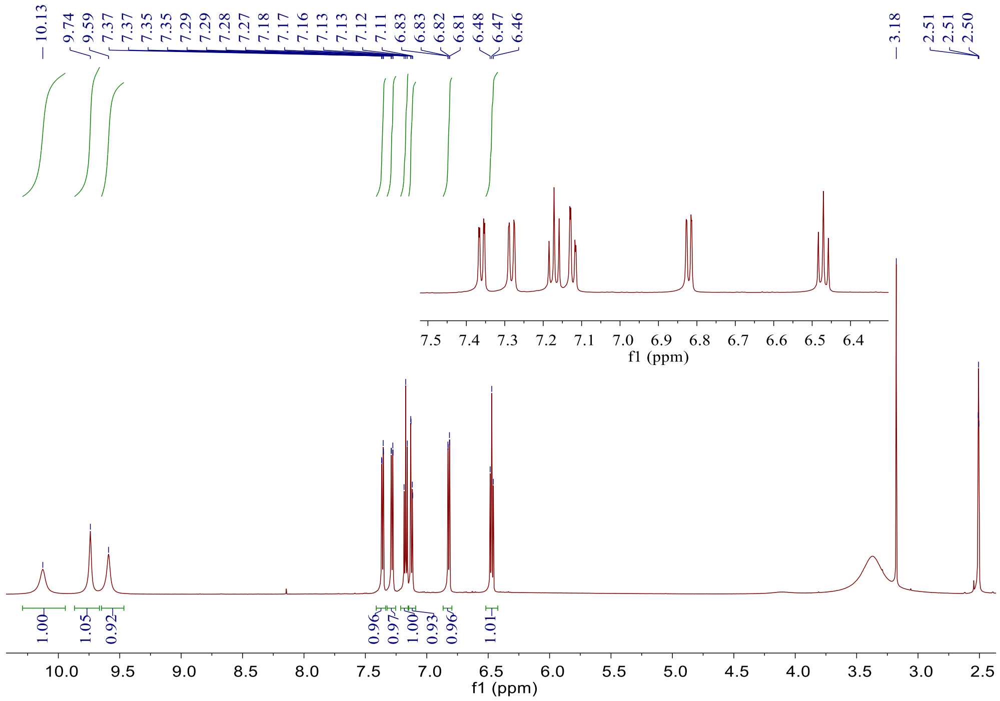
10.13
9.74
9.59
7.37
7.37
7.35
7.35
7.29
7.29
7.28
7.27
7.18
7.17
7.16
7.13
7.13
7.12
7.11
6.83
6.83
6.82
6.81
6.48
6.47
6.46
3.18
2.51
2.51
2.50
1.00
1.05
0.92
0.96
0.97
1.00
0.93
0.96
1.01
f1 (ppm)
f1 (ppm)

b

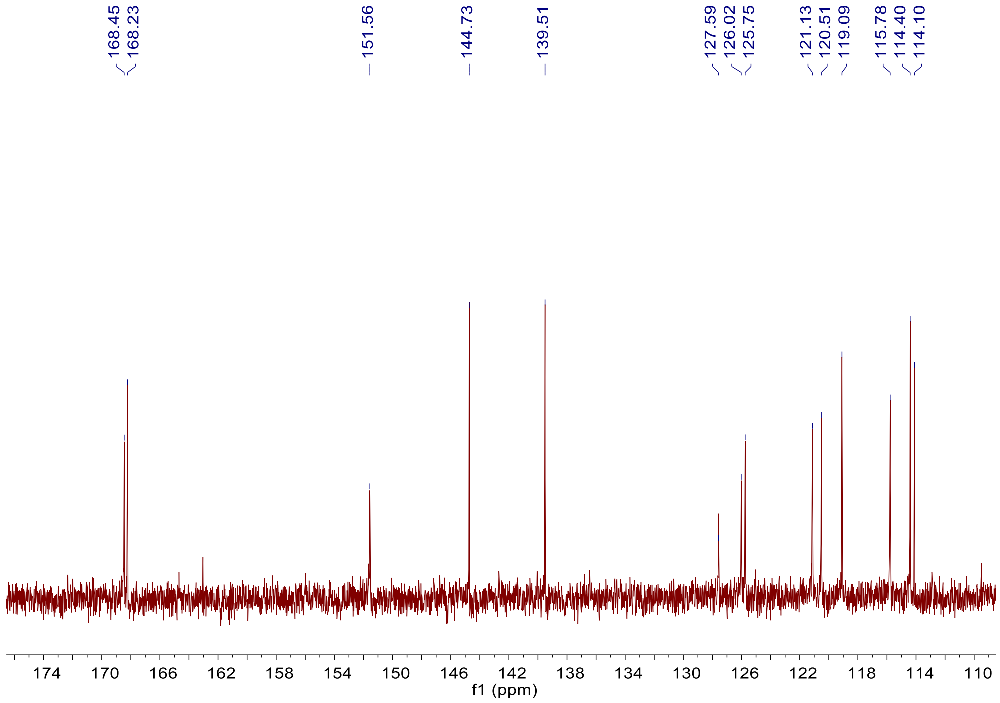
168.45
168.23
151.56
144.73
139.51
127.59
126.02
125.75
121.13
120.51
119.09
115.78
114.40
114.10
f1 (ppm)

c

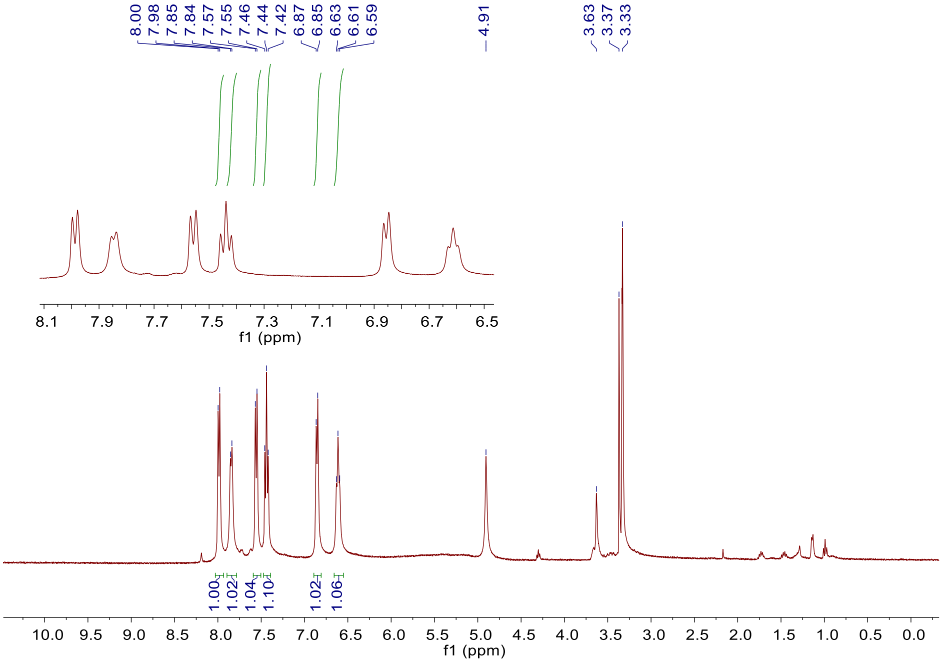

d

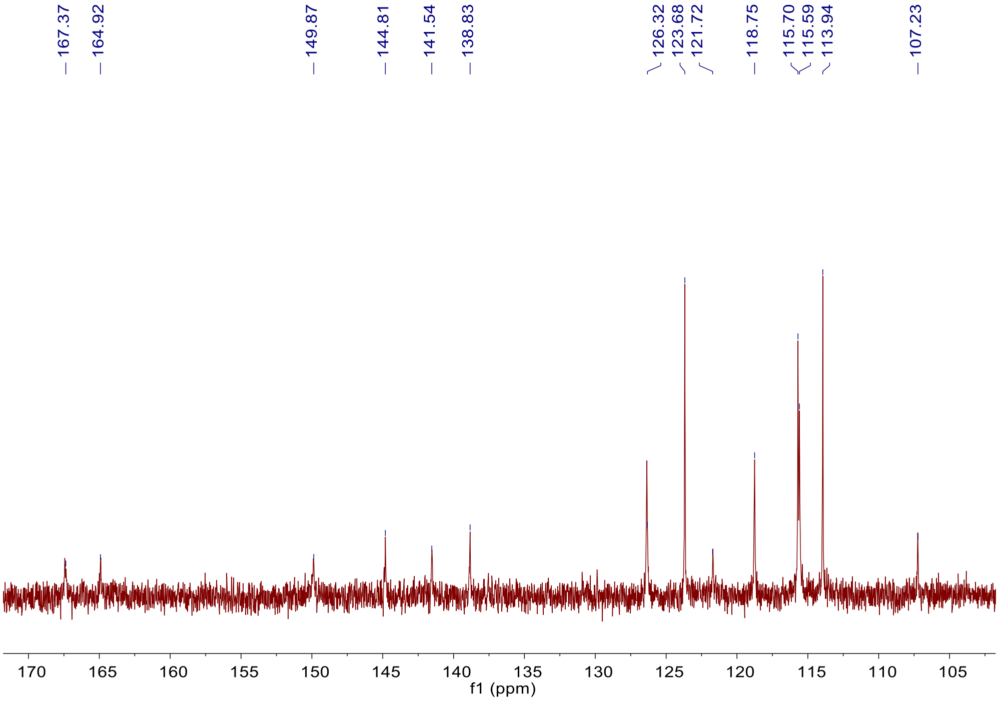

Figure S2.NMR analysis of compound **2 and 3**. **(a)** ^{1}H-NMR spectrum of **2** measured in DMSO-*d*$_6$ at 500 MHz. **(b)** ^{13}C-NMR spectrum of **2** measured in DMSO-*d*$_6$ at 125 MHz. **(c)** ^{1}H-NMR spectrum of **3** measured in CD_3OD at 500 MHz. **(d)** ^{13}C-NMR spectrum of **3** measured in CD_3OD at 125 MHz.

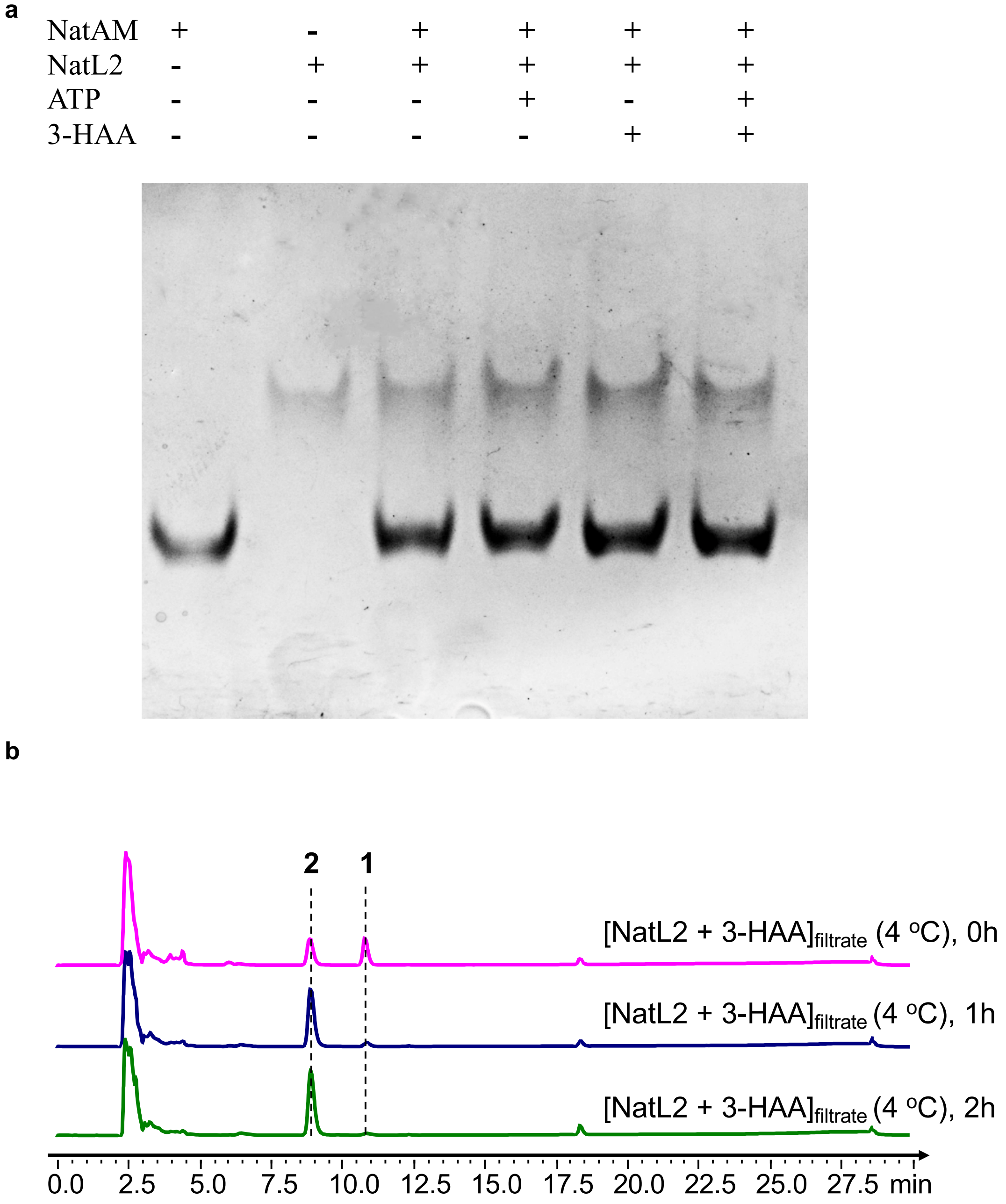

Figure S3. (a) Native page gel electrophoresis of NatL2 and NatAM. Samples were incubated at 30 ºC for 1h in buffer containing 50 mM Tris (pH 8.0) and 10 mM $MgCl_2$ before loading on the gel. Both NatL2 and NatAM were at 4 μM. **(b)** Slow spontaneous conversion of **1** to **2** at 4 ºC. NatL2 reactions in presence of 3-HAA and ATP were centrifuged at 4 ºC and the filtrate further incubated at room temperature for 0h, 1h, and 2h before being injected on LC-MS for analysis.

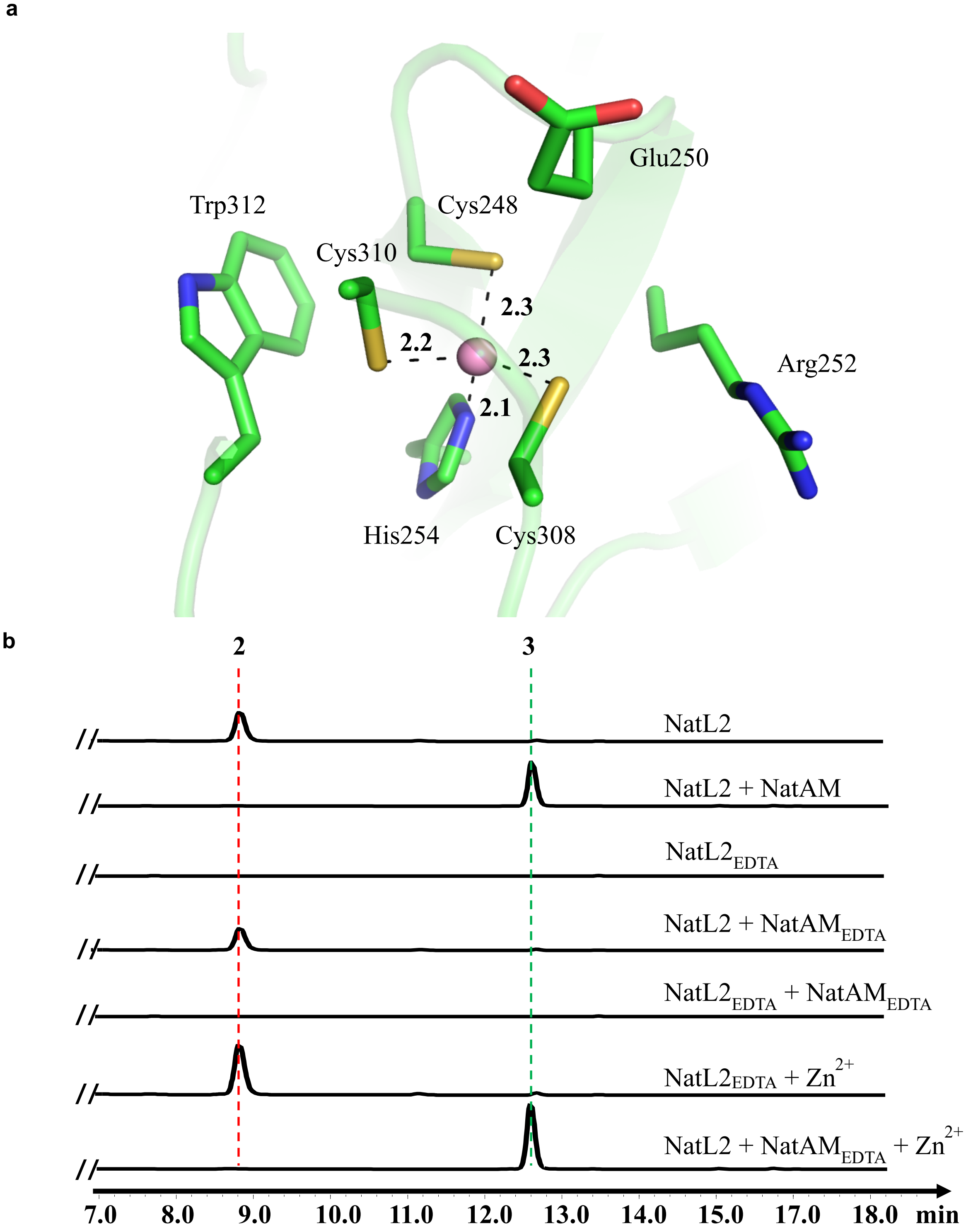

Figure S4. Zinc plays important role for both NatL2 and NatAM. (a) Zinc binding site in NatL2 structures. **(b)** HPLC analysis of the *in vitro* assay of NatL2, EDTA treated NatL2 ($NatL2_{EDTA}$), NatAM and EDTA treated NatAM ($NatAM_{EDTA}$) shows abolition of activity upon treatment with EDTA. Activity is recovered by supplying Zn^{2+}.

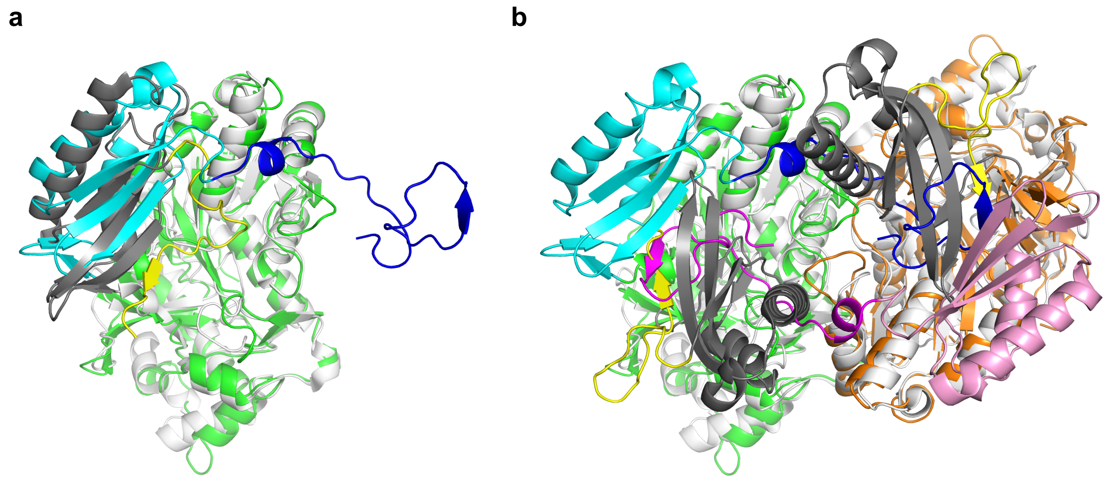

Figure S5. Superimposition of NatL2 with PaaKs in adenylation conformation (2Y27) and thioesterification conformation (2Y4O). **(a).** Monomers of NatL2 and 2Y27 represented as cartoon. NatL2 is colored N-domain green, C-domain cyan and C-terminal extension blue. 2Y27 is colored N-domain white, C-domain grey and C-terminal region yellow **(b)**. Dimers of NatL2 is locked in the adenylation conformation by C-terminal extension, this is very different to the thioesterification conformation of 2Y4O. Monomer B of NatL2 , N orange, C pink and C-extension purple. 2Y4O is colored as 2Y27 in S5a.

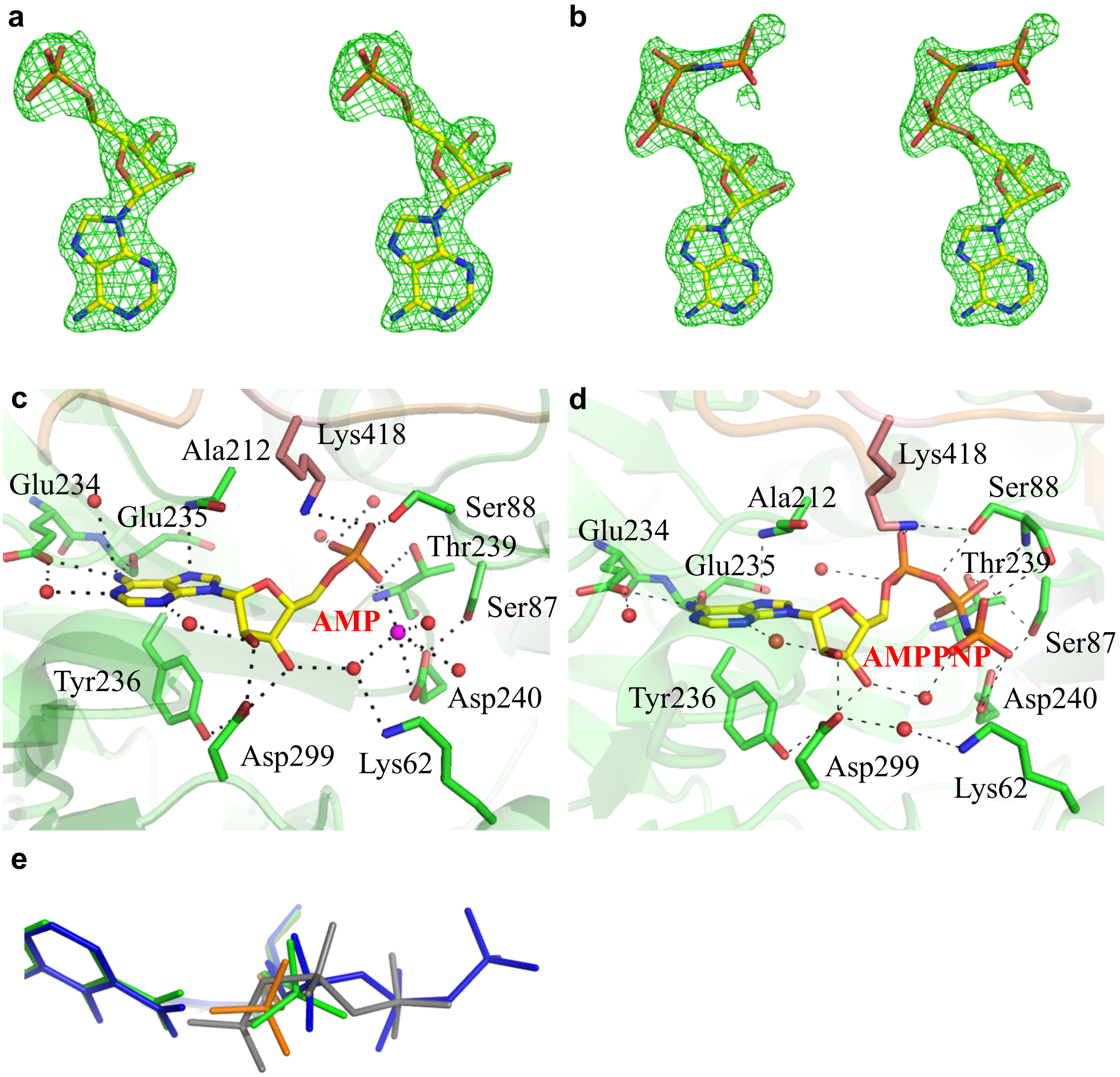

Figure S6. Nucleotide binding site in NatL2 structures. **(a-b).** Stereo view of simulated-annealing Fo-Fc omit electron density (contoured at 2.5 σ) for AMPPNP and AMP in NatL2:AMPPNP and NatL2:AMP structures. **(c-d).** AMPPNP and AMPPNP binding sites for both structures. **(e)** Superimposition of NatL2 structures showing movement of a-phosphate during catalysis. Ligands are colored blue (NatL2:AMPPNP:3-HAA), green (NatL2:AMP:3-HAA), grey (NatL2:AMPPNP), and orange (AMP).

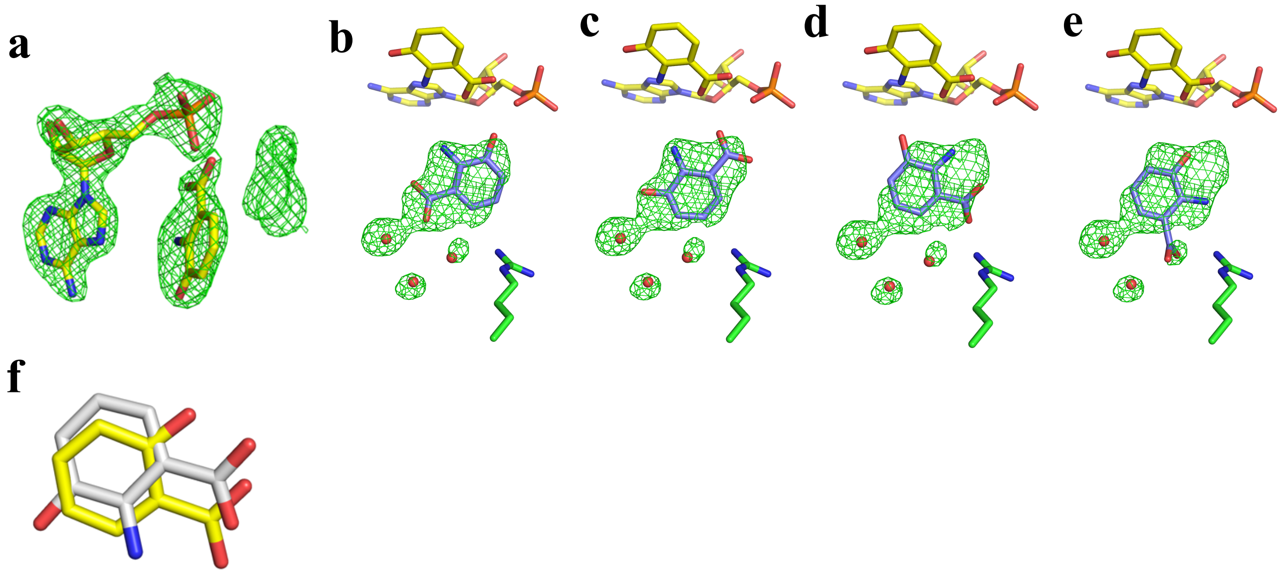

Figure S7. NatL2:AMP:3-HAA and NatL2:SA complex structures. (a) Fo-Fc omit map (3 σ). of AMP, 3-HAA[1], and putative 3-HAA[2]. **(b-e)** Fitting of different orientation of 3-HAA[2] in the electron density (Fo-Fc Omit map contoured at 2.5 σ). The results show (b) gave the best fitting. **(f)** Superimposition of 3-HAA[1] (from NatL2:AMP:3-HAA complex) and SA[1] (from NatL2:SA complex).

a

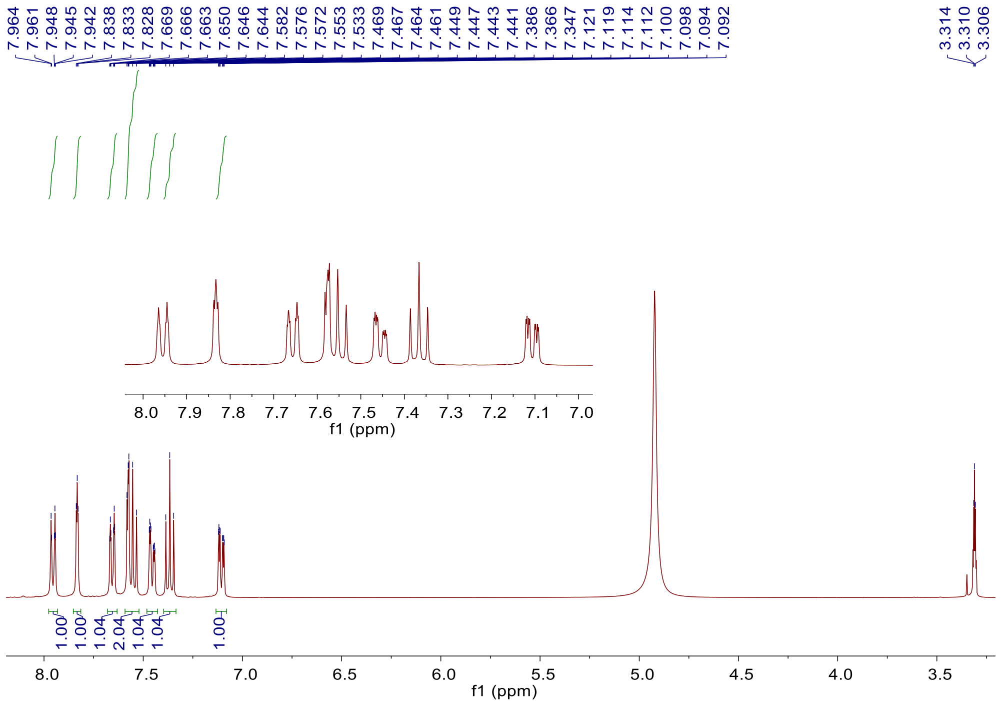
7.964
7.961
7.948
7.945
7.942
7.838
7.833
7.828
7.669
7.666
7.663
7.650
7.646
7.644
7.582
7.576
7.572
7.553
7.533
7.469
7.467
7.464
7.461
7.449
7.447
7.443
7.441
7.386
7.366
7.347
7.121
7.119
7.114
7.112
7.100
7.098
7.094
7.092
3.314
3.310
3.306
1.00
1.00
1.04
2.04
1.04
1.04
1.00
f1 (ppm)
f1 (ppm)

b

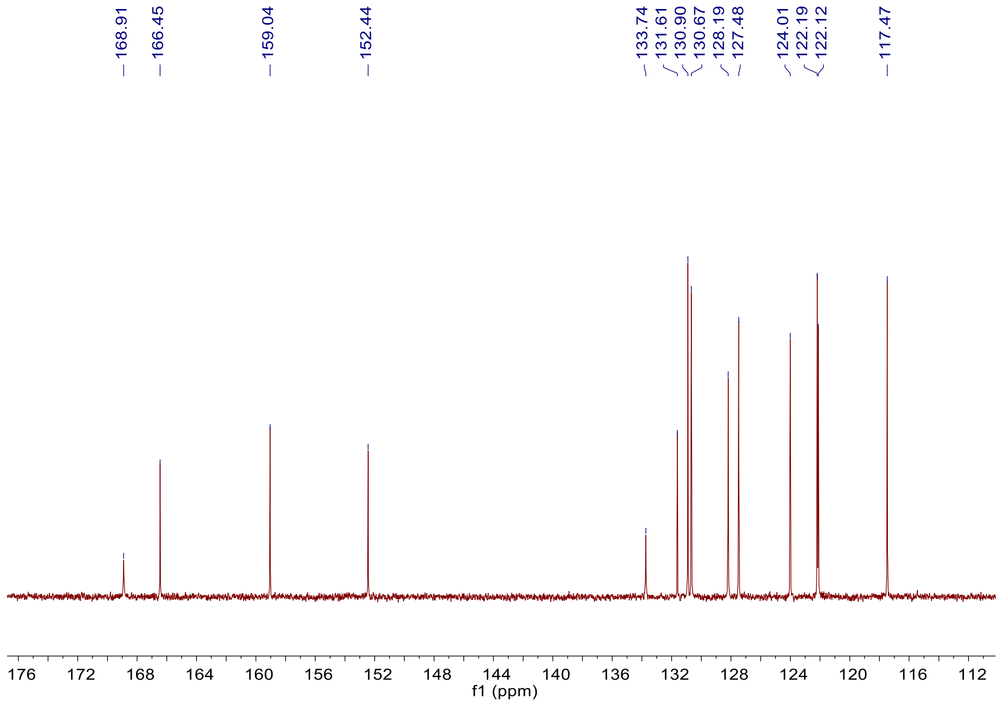
168.91
166.45
159.04
152.44
133.74
131.61
130.90
130.67
128.19
127.48
124.01
122.19
122.12
117.47
f1 (ppm)

c

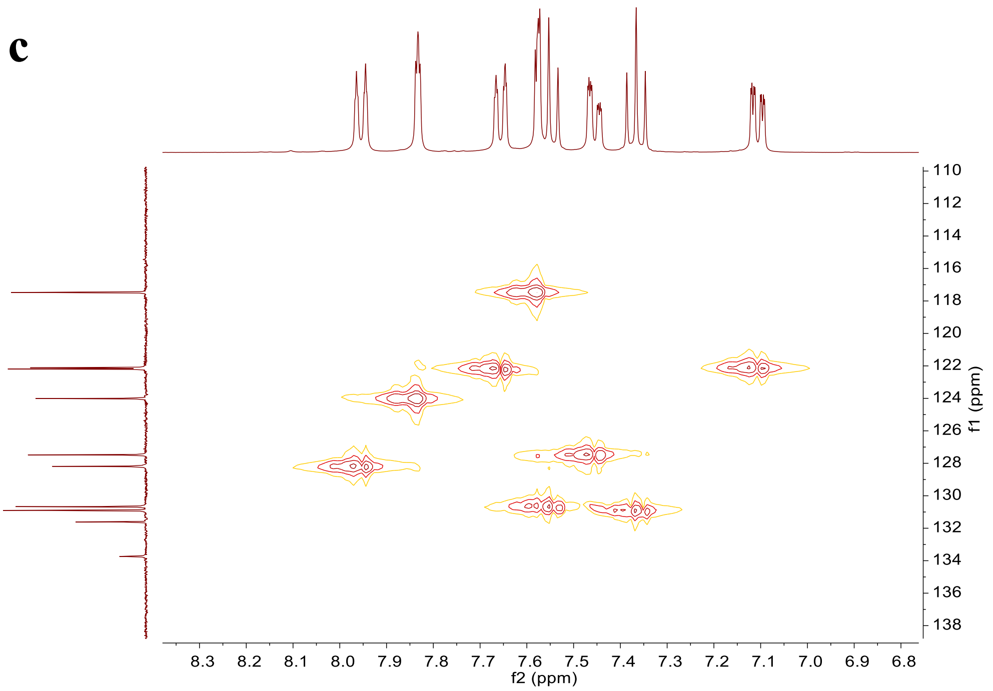

d

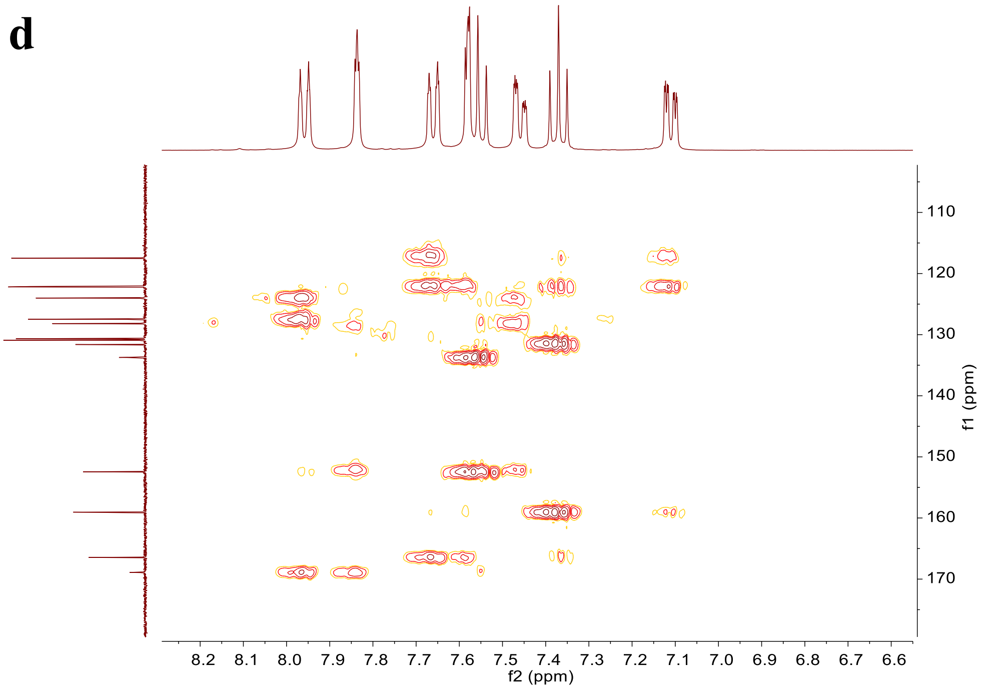

Figure S8. NMR analysis of compound **4**. **(a)** ^{1}H-NMR spectrum of compound **4** measured in CD_3OD at 400 MHz. **(b)** ^{13}C-NMR spectrum of compound **4** measured in CD_3OD at 100 MHz. **(c)** HSQC spectrum of compound **4** measured in CD_3OD at 100 MHz. **(d)** HMBC spectrum of compound **4** measured in CD_3OD at 100 MHz.

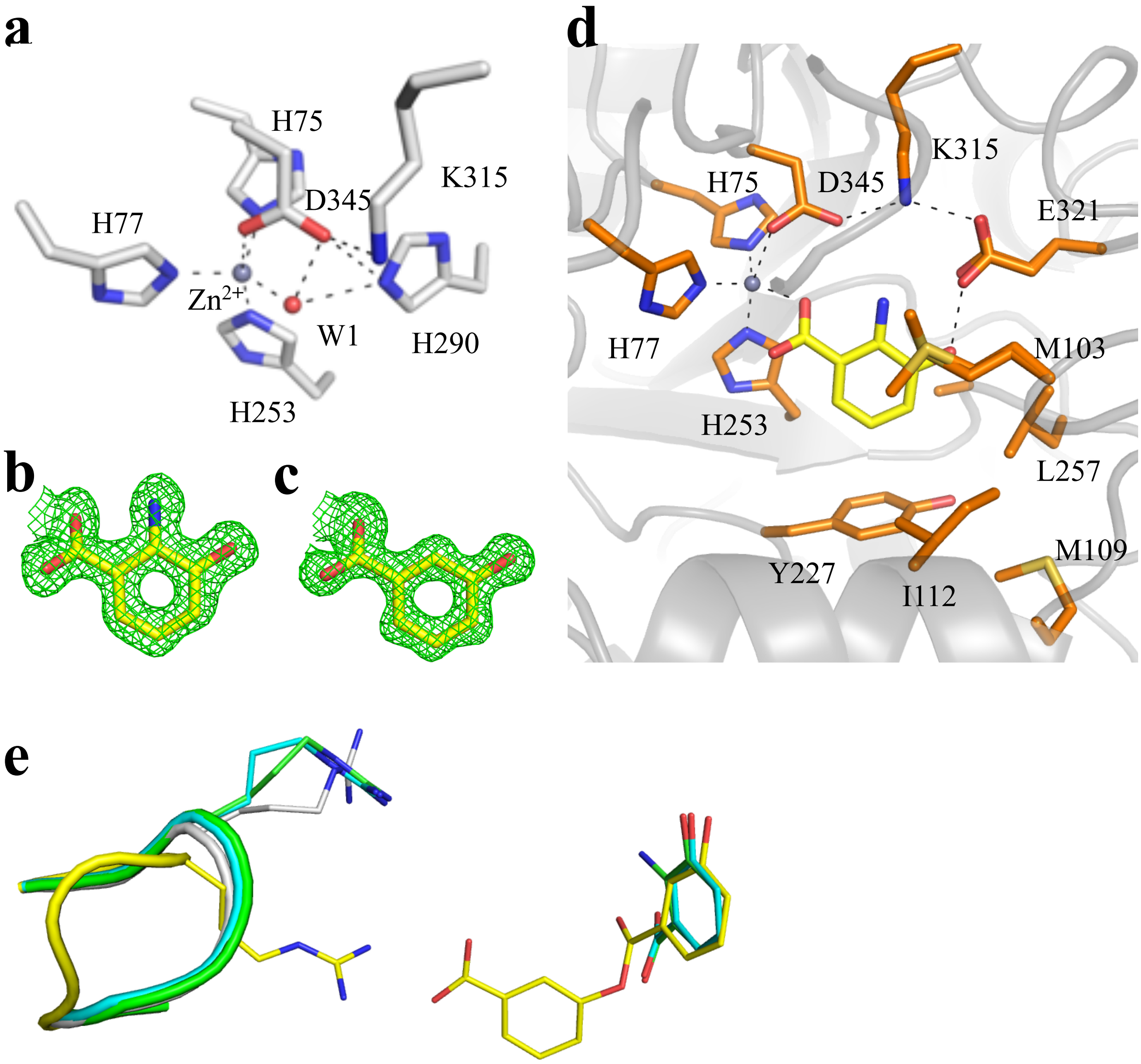

Figure S9. Structures of NatAM. (a) Zinc binding site for apo NatAM structure. Residues were colored with carbon atoms white, nitrogen blue, oxygen red. Zinc ion is shown as grey sphere and water molecule is in red sphere, **(a-b)**. simulated-annealing Fo-Fc omit electron density (contoured at 2.5 σ) of 3-HAA and 3-HBA. **(c).** 3-HAA binding pocket revealed by NatAM:3-HAA structure. **(d).** Superimposition of different NatAM structures shows a loop (Phe84-Gly88) flips upon binding of 4. Loop and ligand carbon atoms are colored yellow (NatAM:**4**), green (NatAM:3-HAA), cyan (NatAM:3-HBA) and white (*apo* NatAM). Arg87 is shown in sticks and colored respectively.

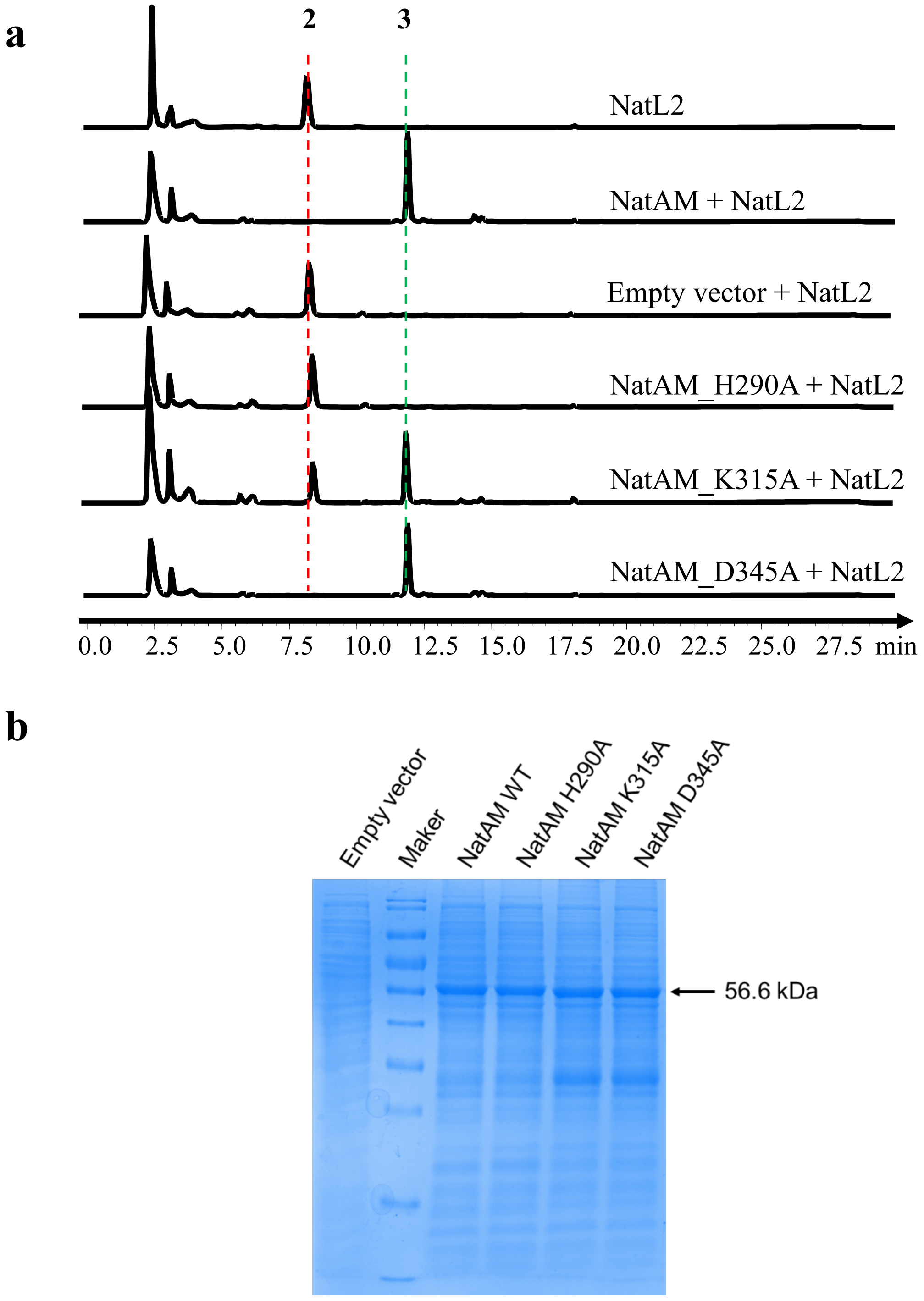

Figure S10. *In vitro* assay of NatAM variants with NatL2. (a) HPLC analysis of the *in vitro* cell-free assay of NatAM mutants and purified NatL2. (b) SDS-PAGE of NatAM mutants. Empty vector was used as a control.

a

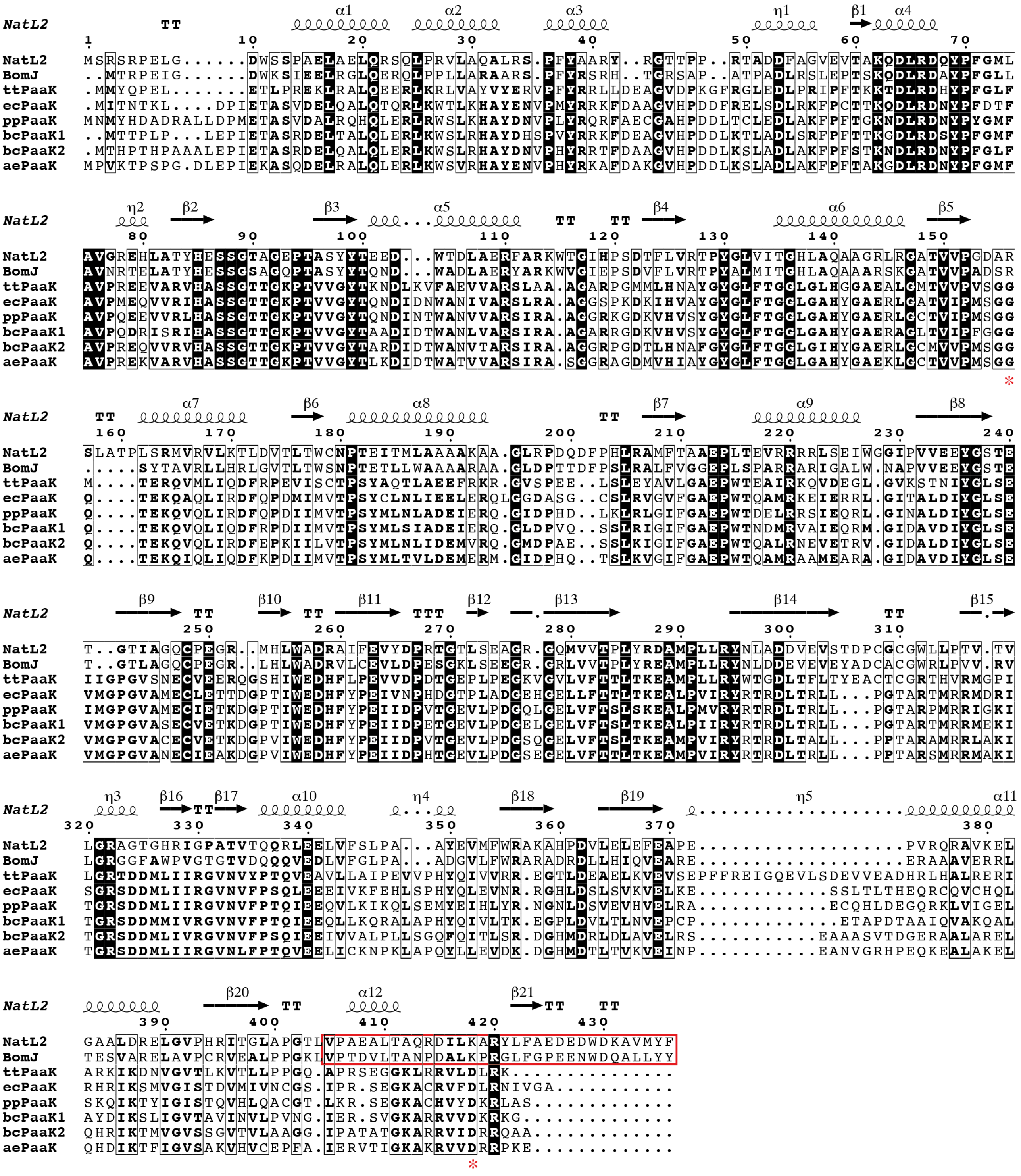
NatL2
α1 α2 α3 η1 β1 α4
1 10 20 30 40 50 60 70
NatL2 MSRSRPELG......DWSSPAELAELQRSQLPRVLAQALRS.PFYAARY..RGTTPP..RTADDFAGVEVTAKQDLRDQYPFGML
BomJ ..MTRPEIG......DWKSIEELRGLQERQLPPLLARAARS.PFYRSRH..TGRSAP..ATPADLRSLEPTSKQDLRDAYPFGLL
ttPaaK .MMYQPEL.......ETLPREKLRALQEERLKRLVAYVYERVPFYRRLLDEAGVDPKGFRGLEDLPRIPFTKKTDLRDHYPFGLF
ecPaaK .MITNTKL....DPIETASVDELQALQTQRLKWTLKHAYENVPMYRRKFDAAGVHPDDFRELSDLRKFPCTTKQDLRDNYPFDTF
ppPaaK MNMYHDADRALLDPMETASVDALRQHQLERLRWSLKHAYDNVPLYRQRFAECGAHPDDLTCLEDLAKFPFTGKNDLRDNYPYGMF
bcPaaK1 ..MTTPLP...LEPIETASRDELTALQLERLKWSLRHAYDHSPVYRRKFDEAGVHPDDLKTLADLSRFPFTTKGDLRDSYPFGMF
bcPaaK2 .MTHPTHPAAALEPIETASRDELQALQLERLKWSLRHAYDNVPHYRRTFDAAGVHPDDLKSLADLAKFPFSTKNDLRDNYPFGLF
aePaaK MPVKTPSPG.DLEPIEKASQDELRALQLERLKWSVRHAYENVPHYRKAFDAKGVHPDDLKSLADLAKFPFTAKGDLRDNYPFGMF
NatL2
η2 β2 β3 α5 TT TT β4 α6 β5
80 90 100 110 120 130 140 150
NatL2 AVGREHLATYHESSGTAGEPTASYYTEED...WTDLAERFARKWTGIHPSDTFLVRTPYGLVITGHLAQAAGRLRGATVVPGDAR
BomJ AVNRTELATYHESSGSAGQPTASYYTQND...WADLAERYARKWVGIEPSDVFLVRTPYALMITGHLAQAAARSKGATVVPADSR
ttPaaK AVPREEVARVHASSGTTGKPTVVGYTKNDLKVFAEVVARSLAA.AGARPGMMLHNAYGYGLFTGGLGLHGGAEALGMTVVPVSGG
ecPaaK AVPMEQVVRIHASSGTTGKPTVVGYTQNDIDNWANIVARSLRA.AGGSPKDKIHVAYGYGLFTGGLGAHYGAERLGATVIPMSGG
ppPaaK AVPQEEVVRLHASSGTTGKPTVVGYTQNDINTWANVVARSIRA.AGGRKGDKVHVSYGYGLFTGGLGAHYGAERLGCTVIPMSGG
bcPaaK1 AVPQDRISRIHASSGTTGKPTVVGYTAADIDTWANLVARSIRA.AGARRGDKVHVSYGYGLFTGGLGAHYGAERAGLTVIPFGGG
bcPaaK2 AVPREQVVRVHASSGTTGKPTVVGYTARDIDTWANVTARSIRA.AGGRPGDTLHNAFGYGLFTGGLGIHYGAERLGCMVVPMSGG
aePaaK AVPREKVARVHASSGTTGKPTVVGYTLKDIDTWATVVARSIRA.SGGRAGDMVHIAYGYGLFTGGLGAHYGAEKLGCTVVPMSGG
*
NatL2
TT α7 β6 α8 TT β7 α9 β8
160 170 180 190 200 210 220 230 240
NatL2 SLATPLSRMVRVLKTLDVTLTWCNPTEITMLAAAAKAA.GLRPDQDFPHLRAMFTAAEPLTEVRRRRLSEIWGGIPVVEEYGSTE
BomJ .....SYTAVRLLHRLGVTLTWSNPTETLLWAAAARAA.GLDPTTDFPSLRALFVGGEPLSPARRARIGALW.NAPVVEEYGSTE
ttPaaK M....TERQVMLIQDFRPEVISCTPSYAQTLAEEFRKR.GVSPEE..LSLEYAVLGAEPWTEAIRKQVDEGL.GVKSTNIYGLSE
ecPaaK Q....TEKQAQLIRDFQPDMIMVTPSYCLNLIEELERQLGGDASG..CSLRVGVFGAEPWTQAMRKEIERRL.GITALDIYGLSE
ppPaaK Q....TEKQVQLIRDFQPDIIMVTPSYMLNLADEIERQ.GIDPHD..LKLRLGIFGAEPWTDELRRSIEQRL.GINALDIYGLSE
bcPaaK1 Q....TEKQVQLIQDFRPDIIMVTPSYMLSIADEIERQ.GLDPVQ..SSLRIGIFGAEPWTNDMRVAIEQRM.GIDAVDIYGLSE
bcPaaK2 Q....TEKQVQLIRDFEPKIILVTPSYMLNLIDEMVRQ.GMDPAE..SSLKIGIFGAEPWTQALRNEVETRV.GIDALDIYGLSE
aePaaK Q....TEKQIQLIQDFKPDIIMVTPSYMLTVLDEMERM.GIDPHQ..TSLKVGIFGAEPWTQAMRAAMEARA.GIDAVDIYGLSE
NatL2
β9 TT β10 TT β11 TTT β12 β13 β14 TT β15
250 260 270 280 290 300 310
NatL2 T..GTIAGQCPEGR..MHLWADRAIFEVYDPRTGTLSEAGR.GQMVVTPLYRDAMPLLRYNLADDVEVSTDPCGCGWLLPTV.TV
BomJ T..GTLAGQCPEGR..LHLWADRVLCEVLDPESGKLSEEGR.GRLVVTPLYREAMPLLRYNLDDEVEVEYADCACGWRLPVV.RV
ttPaaK IIGPGVSNECVEERQGSHIWEDHFLPEVVDPDTGEPLPEGKVGVLVFTTLTKEAMPLLRYWTGDLTFLTYEACTCGRTHVRMGPI
ecPaaK VMGPGVAMECLETTDGPTIWEDHFYPEIVNPHDGTPLADGEHGELLFTTLTKEALPVIRYRTRDLTRLL...PGTARTMRRMDRI
ppPaaK IMGPGVAMECIETKDGPTIWEDHFYPEIIDPVTGEVLPDGQLGELVFTSLSKEALPMVRYRTRDLTRLL...PGTARPMRRIGKI
bcPaaK1 VMGPGVASECVETKDGPTIWEDHFYPEIIDPETGEVLPDGELGELVFTSLTKEALPIIRYRTRDLTRLL...PGTARTMRRMEKI
bcPaaK2 VMGPGVACECVETKDGPVIWEDHFYPEIIDPVTGEVLPDGSQGELVFTSLTKEAMPVIRYRTRDLTALL...PPTARAMRRLAKI
aePaaK VMGPGVANECIEAKDGPVIWEDHFYPEIIDPHTGEVLPDGSEGELVFTTLTKEAMPVIRYRTRDLTRLL...PPTARSMRRMAKI
NatL2
η3 β16 TT β17 α10 η4 β18 β19 η5 α11
320 330 340 350 360 370 380
NatL2 LGRAGTGHRIGPATVTQQRLEELVFSLPA...AYEVMFWRAKAHPDVLELEFEAPE..................PVRQRAVKEL
BomJ LGRGGFAWPVGTGTVDQQQVEDLVFGLPA...ADGVLFWRARADRDLLHIQVEARE..................ERAAAVERRL
ttPaaK LGRTDDMLIIRGVNVYPTQVEAVLLAIPEVVPHYQIVVRR.EGTLDEAELKVEVSEPFFREIGQEVLSDEVVEADHRLHALRERI
ecPaaK SGRSDDMLIIRGVNVFPSQLEEEIVKFEHLSPHYQLEVNR.RGHLDSLSVKVELKE............SSLTLTHEQRCQVCHQL
ppPaaK TGRSDDMLIIRGVNVFPTQIEEQVLKIKQLSEMYEIHLYR.NGNLDSVEVHVELRA............ECQHLDEGQRKLVIGEL
bcPaaK1 TGRSDDMMIVRGVNVFPTQIEEQLLKQRALAPHYQIVLTK.EGPLDVLTLNVEPCP............ETAPDTAAIQVAKQAL
bcPaaK2 TGRSDDMLIVRGVNVFPSQIEEIVVALPLLSGQFQITLSR.DGHMDRLDLAVELRS............EAAASVTDGERAALAREL
aePaaK TGRSDDMLIIRGVNLFPTQVEELICKNPKLAPQYLLEVDK.DGHMDTLTVKVEINP............EANVGRHPEQKEALAKEL
NatL2
β20 TT α12 β21 TT TT
390 400 410 420 430
NatL2 GAALDRELGVPHRITGLAPGTLVPAEALTAQRDILKARYLFAEDEDWDKAVMYF
BomJ TESVARELAVPCRVEALPPGKLVPTDVLTANPDALKPRGLFGPEENWDQALLYY
ttPaaK ARKIKDNVGVTLKVTLLPPGQ.APRSEGGKLRRVLDLRK..............
ecPaaK RHRIKSMVGISTDVMIVNCGS.IPR.SEGKACRVFDLRNIVGA...........
ppPaaK SKQIKTYIGISTQVHLQACGT.LKR.SEGKACHVYDKRLAS.............
bcPaaK1 AYDIKSLIGVTAVINVLPVNG.IER.SVGKARRVVDKRKG..............
bcPaaK2 QHRIKTMVGVSSGVTVLAAGG.IPATATGKARRVIDRRQAA.............
aePaaK QHDIKTFIGVSAKVHVCEPFA.IERVTIGKAKRVVDRRPKE.............
*

b

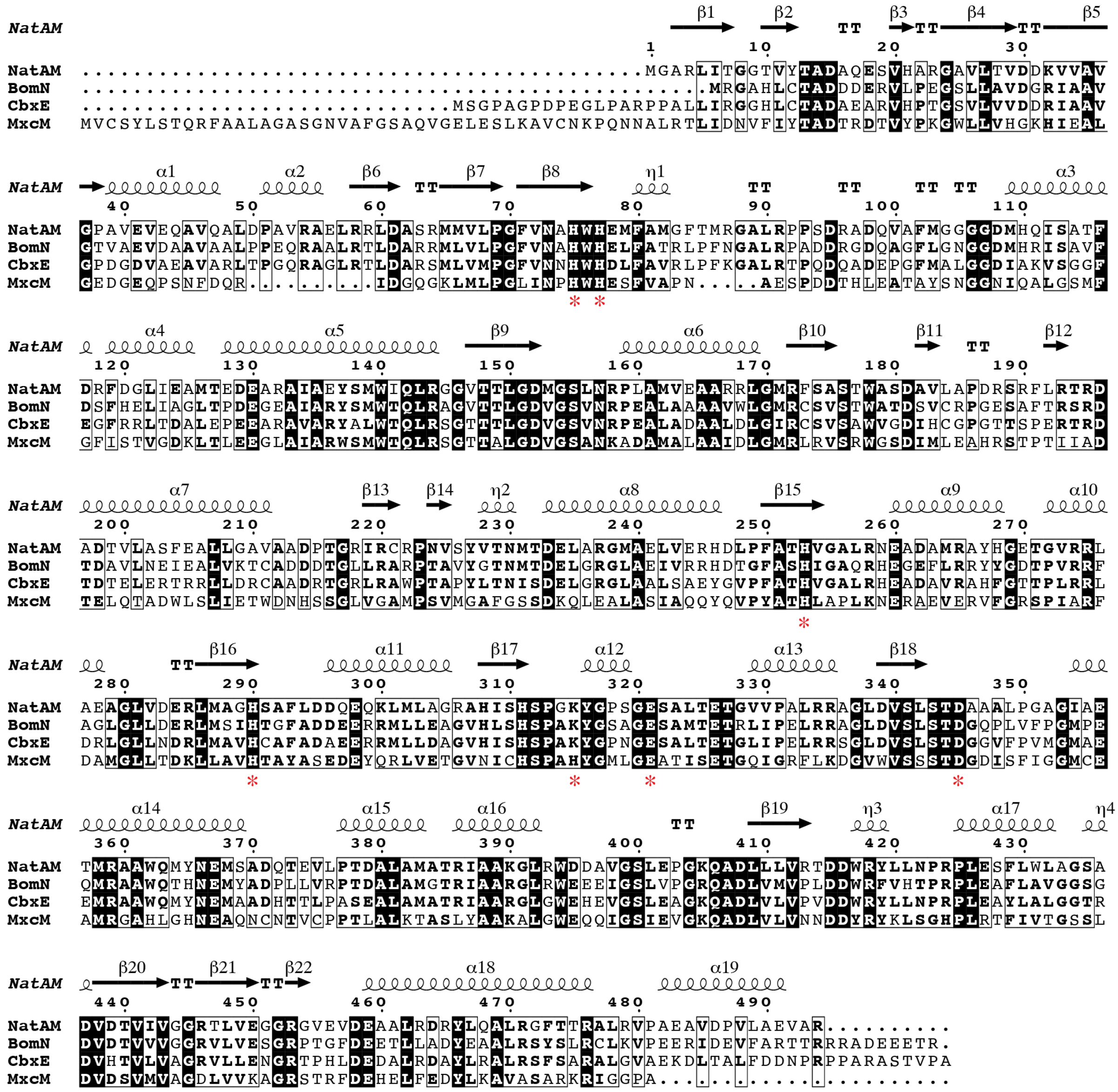

Figure S11. Sequence alignments of NatL2 and NatAM. (a) Sequence alignment of NatL2, BomJ and selected Paaks. The C-terminal crossing motif in both NatL2 and BomJ is highlighted by red box. K418 and R156 of NatL2 are marked with asterisk at the bottom. Abbreviations for the strains of selected Paaks are as follows: tt, *Thermus thermophilus*; ec, *Escherichia coli*; pp, *Pseudomonas putida*; bc, *Burkholderia cenocepacia*; ae, *Azoarcus evansii.* (b) Sequence alignment of NatAM, and related amidohydrolases catalyzing heterocyclization. The residues involved in zinc binding and catalysis are marked with asterisk at the bottom.

Alignments were prepared with MUSCLE and was rendered with ESPript 3.0 with secondary structure of NatL2 or NatAM depicted above the sequence alignment. In both (a) and (b) a black box with white text denotes strict identity, a black bold character shows sequence similarity and black box highlights conserved regions.

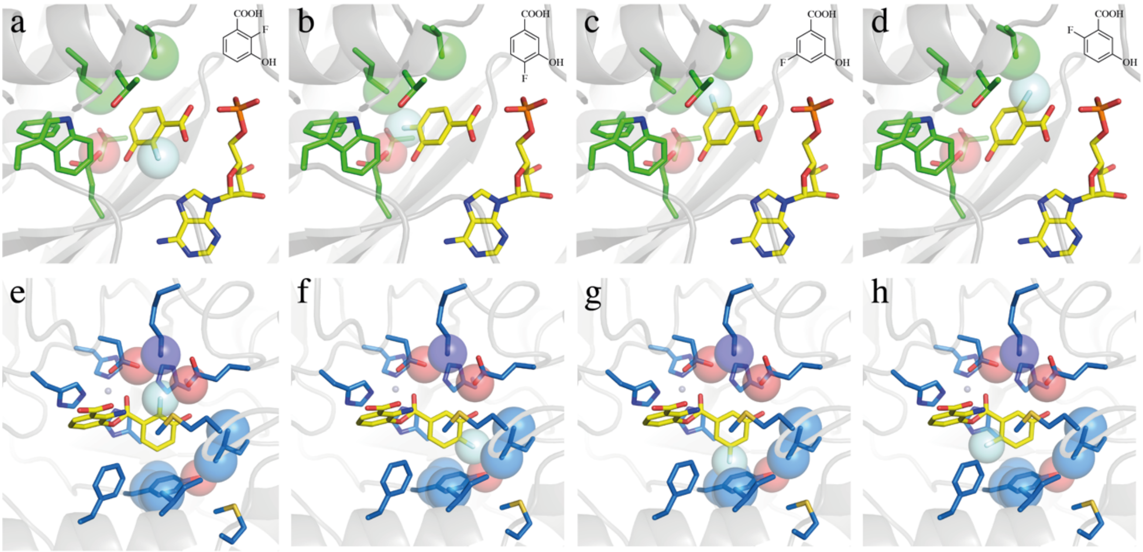

Figure S12. Modelling of 4 fluorine substituted 3-HBAs HAA at the active site of NatL2 (top) and the predicted ester with 3-HBA at the NatAM active site (bottom).

a

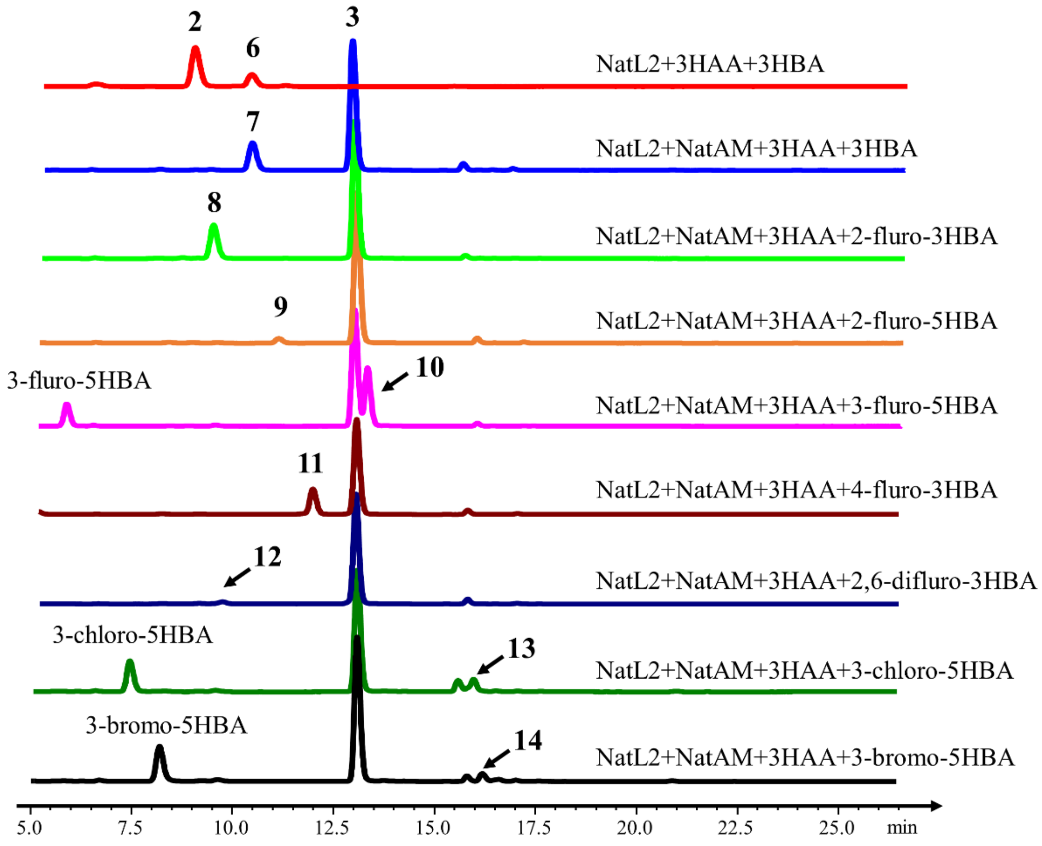
2
6
3
NatL2+3HAA+3HBA
7
NatL2+NatAM+3HAA+3HBA
8
NatL2+NatAM+3HAA+2-fluro-3HBA
9
NatL2+NatAM+3HAA+2-fluro-5HBA
3-fluro-5HBA
10
NatL2+NatAM+3HAA+3-fluro-5HBA
11
NatL2+NatAM+3HAA+4-fluro-3HBA
12
NatL2+NatAM+3HAA+2,6-difluro-3HBA
3-chloro-5HBA
13
NatL2+NatAM+3HAA+3-chloro-5HBA
3-bromo-5HBA
14
NatL2+NatAM+3HAA+3-bromo-5HBA
5.0
7.5
10.0
12.5
15.0
17.5
20.0
22.5
25.0
min

b

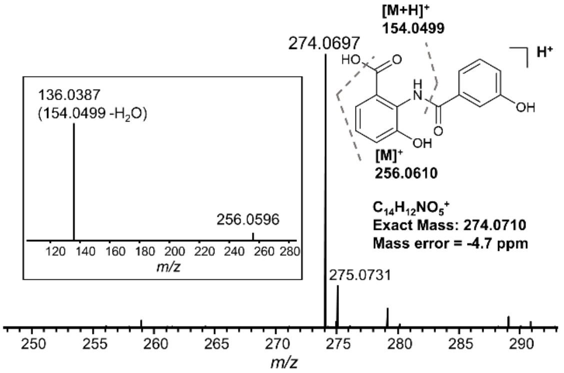

Compound 6

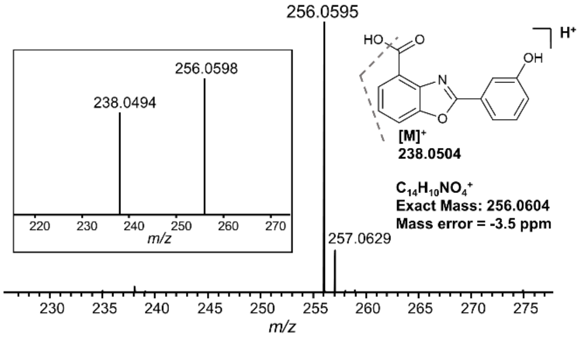

Compound 7

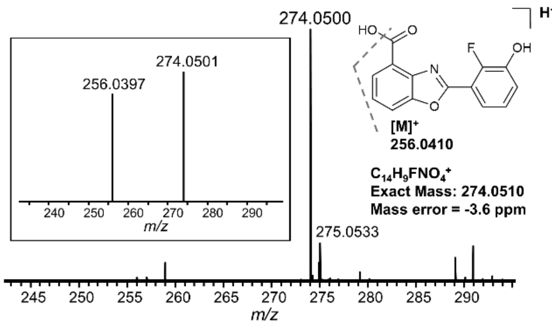

Compound 8

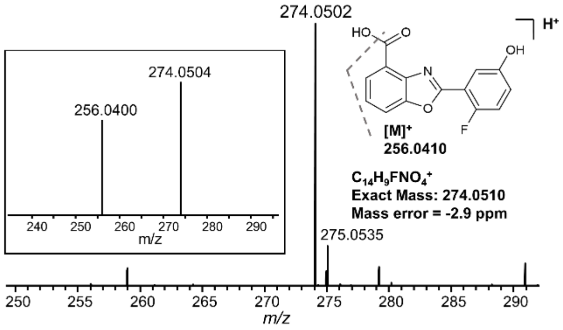

Compound 9

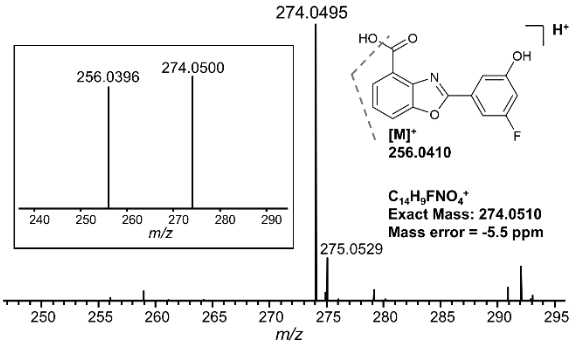

Compound 10

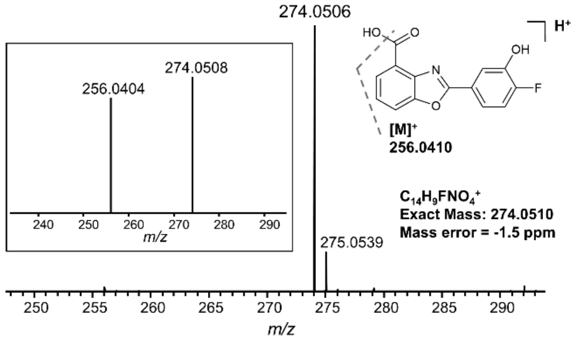

Compound 11

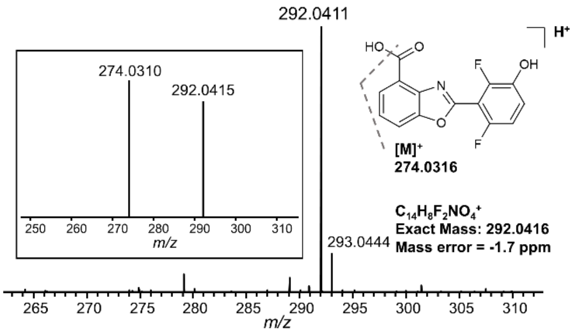

Compound 12

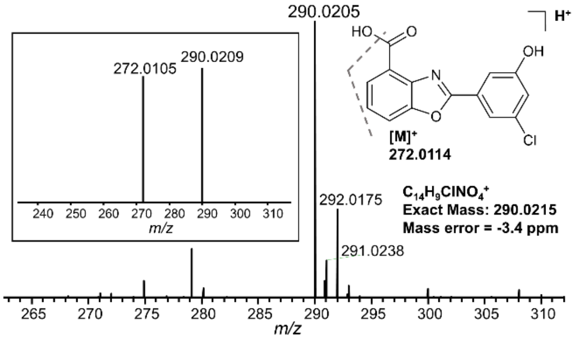

Compound 13

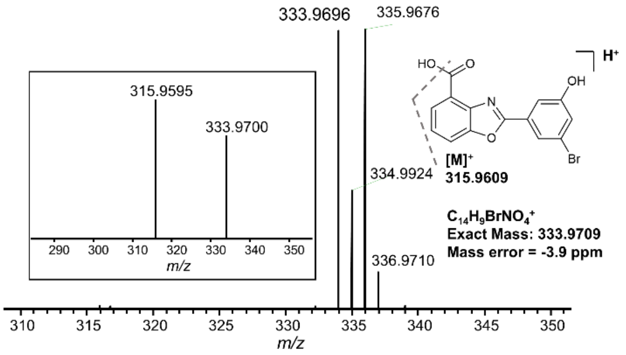

Compound 14

Figure S13. *In vitro* assays of NatL2 and NatAM to explore substrate scope. **(a)** HPLC analysis showing new product formation in the reactions containing NatL2, NatAM, 3-HAA and various halogenated 3-HBAs. **(b)** MS spectrum and MS/MS analysis of compounds **6**-**14** observed in HPLC.

References

[1] M. Lv, X. Ji, J. Zhao, Y. Li, C. Zhang, L. Su, W. Ding, Z. Deng, Y. Yu, Q. Zhang, *J. Am. Chem. Soc.* **2016**, *138*, 6427–6435.
[2] G. Winter, *J Appl Crystallogr* **2010**, *43*, 186–190.
[3] G. Winter, D. G. Waterman, J. M. Parkhurst, A. S. Brewster, R. J. Gildea, M. Gerstel, L. Fuentes-Montero, M. Vollmar, T. Michels-Clark, I. D. Young, et al., *Acta Crystallogr. D Struct. Biol.* **2018**, *74*, 85–97.
[4] C. Vonrhein, C. Flensburg, P. Keller, A. Sharff, O. Smart, W. Paciorek, T. Womack, G. Bricogne, *Acta Crystallogr. Sect. D, Biol. Crystallogr.* **2011**, *67*, 293–302.
[5] C. Vonrhein, E. Blanc, P. Roversi, G. Bricogne, *Methods Mol. Biol.* **2007**, *364*, 215–230.
[6] G. Langer, S. X. Cohen, V. S. Lamzin, A. Perrakis, *Nat. Protoc.* **2008**, *3*, 1171–1179.
[7] T. C. Terwilliger, R. W. Grosse-Kunstleve, P. V. Afonine, N. W. Moriarty, P. H. Zwart, L. W. Hung, R. J. Read, P. D. Adams, *Acta Crystallogr. Sect. D, Biol. Crystallogr.* **2008**, *64*, 61–69.
[8] G. N. Murshudov, A. A. Vagin, E. J. Dodson, *Acta Crystallogr. Sect. D, Biol. Crystallogr.* **1997**, *53*, 240–255.
[9] P. D. Adams, P. V. Afonine, G. Bunkóczi, V. B. Chen, I. W. Davis, N. Echols, J. J. Headd, L.-W. Hung, G. J. Kapral, R. W. Grosse-Kunstleve, et al., *Acta Crystallogr. Sect. D, Biol. Crystallogr.* **2010**, *66*, 213–221.
[10] P. Emsley, B. Lohkamp, W. G. Scott, K. Cowtan, *Acta Crystallogr. Sect. D, Biol. Crystallogr.* **2010**, *66*, 486–501.
[11] R. P. Joosten, F. Long, G. N. Murshudov, A. Perrakis, *IUCrJ* **2014**, *1*, 213–220.
[12] A. J. McCoy, R. W. Grosse-Kunstleve, P. D. Adams, M. D. Winn, L. C. Storoni, R. J. Read, *J Appl Crystallogr* **2007**, *40*, 658–674.
[13] S. McNicholas, E. Potterton, K. S. Wilson, M. E. M. Noble, *Acta Crystallogr. Sect. D, Biol. Crystallogr.* **2011**, *67*, 386–394.
[14] R. P. Joosten, T. A. H. te Beek, E. Krieger, M. L. Hekkelman, R. W. W. Hooft, R. Schneider, C. Sander, G. Vriend, *Nucleic Acids Res.* **2011**, *39*, D411-9.
[15] R. C. Edgar, *Nucleic Acids Res.* **2004**, *32*, 1792–1797.
[16] M. A. Larkin, G. Blackshields, N. P. Brown, R. Chenna, P. A. McGettigan, H. McWilliam, F. Valentin, I. M. Wallace, A. Wilm, R. Lopez, et al., *Bioinformatics* **2007**, *23*, 2947–2948.
[17] X. Robert, P. Gouet, *Nucleic Acids Res.* **2014**, *42*, W320-4.
